# Lead Validation of the Olive Phenolic *S*-(−)-Oleocanthal for Effective Control of *KRAS^G13D^*-Mutant Colorectal Cancer Progression and Metastasis

**DOI:** 10.3390/nu18162623

**Published:** 2026-08-11

**Authors:** Md Towhidul Islam Tarun, Hassan Y. Ebrahim, Khalid A. El Sayed

**Affiliations:** 1 School of Basic Pharmaceutical and Toxicological Sciences, College of Pharmacy, University of Louisiana at Monroe, 1800 Bienville Drive, Monroe, LA 71201, USA; tarunmt@warhawks.ulm.edu; 2 Department of Biomedical Sciences, Discipline of Pharmacology, Edward Via College of Osteopathic Medicine, Monroe, LA 71203, USA; hebrahim@ulm.vcom.edu

**Keywords:** colorectal cancer, extra-virgin olive oil, fecal microbiota transplantation, gut microbiota, HCT-116, *KRAS^G13D^*-mutant, *S*-(−)-Oleocanthal

## Abstract

**Background/Objectives:** Colorectal cancer (CRC) is the second most common cancer-causing death in the United States. The Mediterranean diet, rich in extra-virgin olive oil (EVOO), is associated with a lower risk of colorectal cancer (CRC). Earlier studies reported *S*-(−)-oleocanthal (OC), the major EVOO phenolic, to be effective in CRC progression and recurrence suppression in a subcutaneous xenograft model by targeting the SMYD2–EZH2/c-MET signaling axis and favorably modulating gut microbiota (GM). However, lead validation in an orthotopically xenografted model that mimics the tumor microenvironment is yet to be achieved. The GM contribution to OC anti-CRC activity remains unknown. **Methods:** We used an orthotopic intra-cecally xenografted nude mouse model to validate OC anti-*KRAS*-mutant CRC activity and assessed the contribution of OC GM modulation to this activity using an oral antibiotic depletion strategy. **Results:** Daily oral 10 mg/kg OC impressively suppressed *KRAS^G13D^*-mutant CRC HCT-116-Luc progression and metastasis more effectively than intraperitoneal administration 3×/week. By contrast, GM depletion with a broad-spectrum antibiotics cocktail (ABC) partially attenuated this activity, suggesting GM contribution to OC anti-CRC activity. Thus, fecal microbiota transplantation (FMT) was conducted using fresh daily oral fecal GM treatments collected from 20 mg/kg OC-dosed nude mouse donors. A week before FMT dosing, orthotopic HCT-116-Luc tumor-bearing recipient mice were subjected to GM depletion using daily oral ABC dosing. Recipient mice treated with FMT from OC-treated donors exhibited dramatic >99% reductions in primary and near-complete suppression of multi-organ metastatic tumor burden versus FMT controls. **Conclusions:** Collectively, oral OC is validated as an effective anti-*KRAS^G13D^*-mutant CRC lead. Future clinical trials can validate its anti-CRC potential in a human model.

## 1. Introduction

Colorectal cancer (CRC) is the second leading cause of cancer-related mortality in the United States and remains a major global health challenge, with an estimated 158,850 new CRC cases and 28,900 and 24,000 deaths anticipated among males and females, respectively, in the United States for 2026 [1,2]. CRC has become the leading cancer associated with mortality in both genders below the age of 50 in the United States [2]. Approximately 20% of patients with CRC are diagnosed with metastatic disease, and up to 50% of those initially diagnosed with localized disease eventually develop metastases, resulting in a 5-year survival rate below 20% [2,3].

Activating mutations in the *KRAS* (Kirsten rat sarcoma viral proto-oncogene) gene represent among the most clinically significant molecular alterations in CRC, occurring in 40–50% of cases. Codons 12 and 13 constitute the primary mutation hotspots, collectively accounting for approximately 82% of all KRAS mutations in CRC, while codons 61, 117, and 146 are less frequently affected [4]. These activating mutations confer constitutive activation of downstream oncogenic pathways including MAPK/ERK and PI3K/AKT, driving sustained tumor proliferation, disease progression, poor prognosis, and resistance to anti-EGFR-targeted therapies [5]. Among individual variants, the *KRAS^G12C^* mutation represents ~11% of *KRAS* mutations in CRC, and is only common in 3% of overall CRC cases [6], whereas the *KRAS^G13D^* mutation constitutes a clinically relevant subtype that may exhibit differential responsiveness to anti-EGFR therapy compared with other *KRAS*-mutant subtypes [7]. Combining the *KRAS*^G12C^ inhibitor adagrasib with EGFR-directed cetuximab showed clinical success; however, effective *KRAS*^G13D^ inhibitors are yet to be discovered [6,7]. Single-agent kinase inhibitors, including MEK- and EGFR-targeted therapies, are ineffective against CRCs that harbor *KRAS* mutations. Clinical responses to current *KRAS*-targeted inhibitors are usually incomplete, justifying the need to develop more effective therapies for KRAS-mutant CRC.

Epigenetic dysregulation encompassing aberrant DNA methylation, post-translational histone and non-histone modifications, and chromatin remodeling has been established as a fundamental hallmark of CRC pathogenesis. Among the most extensively studied epigenetic regulators in CRC are the lysine methyltransferases SET and MYND domain-containing protein 2 (SMYD2) and Enhancer of Zeste Homolog 2 (EZH2), both of which have been implicated as key oncogenic drivers [8]. Lysine methylation by EZH2 and SMYD2 modifies the protein substrates bulkiness, hydrophobicity, and molecular recognition by methyl-lysine readers [8,9]. SMYD2 directly methylates EZH2 at lysine residue 307 (K307), potentiating its oncogenic function and driving tumor progression [10,11,12]. Adenomatous polyposis coli 2 (APC2) is a Wnt/β-catenin pathway inhibitor [11]. SMYD2 dysregulation suppresses APC2, activating the Wnt/β-catenin pathway in CRC [11]. SMYD2 has been demonstrated to facilitate tumorigenesis, cellular proliferation, tumor invasion, and chemotherapeutic resistance in multiple gastrointestinal malignancies; moreover, its amplification has been reported across a broad spectrum of human cancer types [8,9,10,11,12,13]. In CRC, SMYD2 expression is significantly elevated in tumor tissues relative to tumor-free controls in both human and murine models [14]. SMYD2 genetic suppression markedly impaired tumor growth in independent experimental systems [14]. Clinically, SMYD2 overexpression in CRC correlated with poor prognosis in patients receiving oxaliplatin-based chemotherapy, and its pharmacological or genetic suppression restored sensitivity to oxaliplatin in CRC cells [9]. Similarly, EZH2 is aberrantly dysregulated in CRC, promoting invasion and metastasis [15,16,17] and modulating macrophage polarization within the tumor microenvironment [16]. Genomic profiling further corroborated the specificity of this oncogenic axis, demonstrating that SMYD2, EZH2, and SMYD3 are aberrantly dysregulated in CRC relative to adjacent normal tissue, whereas SMYD1 and EZH1 exhibit comparatively low expression [11]. Clinically, the concurrent aberrant expressions of SMYD2 and EZH2 are associated with enhanced tumor aggressiveness and adverse patient outcomes in invasive CRC [8].

Current standard-of-care treatments for CRC include resection surgery, systemic chemotherapies, immunotherapies, and radiotherapy, which can extend patient survival. However, they are frequently associated with substantial toxicity, impairment of normal tissue integrity, modulated immune functions, and the progressive development of therapeutic resistance [18]. These shortcomings underscore the urgent need for novel, mechanistically distinct, and better-tolerated therapeutic modalities. Over the past decade, GM has emerged as a critical determinant of CRC pathogenesis and treatment response [19]. Microbial dysbiosis disrupts intestinal homeostasis and contributes to CRC initiation and progression [20,21]. By contrast, GM-targeted interventions, most notably FMT, have demonstrated the capacity to remodel tumor-associated microbial communities and have been evaluated as adjunctive strategies in CRC management [22]. The identification of specific GM-derived biomarkers, including *Fusobacterium nucleatum*, further supports the diagnostic and therapeutic relevance of GM profiling in CRC [23].

Extra-virgin olive oil (EVOO) and its bioactive minor phenolic constituents have attracted growing scientific interest as dietary agents with chemopreventive and anticancer potential. The bioavailability and site of action of EVOO phenolics are governed by their structural complexity, whereby simpler EVOO phenolic primary alcohols such as tyrosol and hydroxytyrosol undergo rapid absorption in the small intestine, while the structurally complex mono- and polyphenols may reach the large intestine and engage in direct crosstalk interactions with the colonic GM [24]. EVOO phenolics–GM interactions modulate the production of GM-derived short-chain fatty acids, with downstream effects on cholesterol trafficking, systemic immune regulation, and energy homeostasis [24]. EVOO consumption has been shown to enhance GM alpha diversity in preclinical models [24]. EVOO, in appropriate dietary contexts, can remodel colonic microbial composition toward potentially chemoprotective configurations [25]. The biological consequences of these shifts are attributable, at least in part, to the production of bioactive GM metabolites and their associated microbial metabolic products with anticancer properties [26]. Clinical data from Mediterranean diet interventions enriched with EVOO demonstrate increased abundance of beneficial gut taxa, including *Bifidobacterium animalis* and the butyrate-producing bacteria *Roseburia faecis*, *Ruminococcus bromii*, and *Oscillospira plautii* [27,28].

*S*-(−)-Oleocanthal (OC), a dialdehydic monophenolic secoiridoid uniquely occurring in EVOO, exerts pleiotropic anticancer activity through mechanisms encompassing oncogenic signaling inhibition, metabolic reprogramming, GM modulation, and precision oncology applications [29,30,31,32,33,34,35,36]. The previous investigations of this study’s team demonstrated that OC potently suppressed subcutaneously xenografted CRC progression and recurrence by targeting the SMYD2–EZH2 epigenetic signaling axis and c-MET activation, both in vitro and in vivo in nude mice [8]. Moreover, employing an ex vivo fecal fermentation model, OC was shown to significantly modulate GM ecology, suppressing 56 bacterial species while increasing the abundance of 28 others [36]. Notwithstanding these findings, whether the anti-CRC efficacy of OC is mediated, in whole or in part, through its modulation of the GM has remained an unresolved mechanistic question.

The present study systematically investigates the contribution of OC-modulated GM to its anti-CRC activity using an oral broad-spectrum antibiotics depletion strategy in an orthotopic intra-cecally xenograft nude mouse model with *KRAS*-mutant CRC. To directly assess the anti-CRC potential of OC-modulated GM, FMT was performed using fresh fecal GM collected from high OC-dosed healthy donor nude mice in nude mice recipients xenografted with aggressive *KRAS^G13D^*-mutant HCT-116-Luc cells; prior to this, they underwent GM depletion using daily oral ABC dosing. Thus, this study validates OC as a potential lead to control *KRAS^G13D^*-mutant CRC.

## 2. Materials and Methods

### 2.1. Chemicals and Reagents

All reagents and chemicals were obtained from Avantor (Radnor, PA, USA) unless otherwise stated. OC was isolated from the Greek Governor EVOO (The Governor, Corfu, Greece) using our previously established liquid–liquid extraction method followed by the SP70 entrapment resin [37]. The OC-enriched fraction was eluted with acetone and further purified by Sephadex LH20 size-exclusion column chromatography using CH_2_Cl_2_ with stepwise-controlled increases in EtOAc. The final product achieved ≥99% purity, which was confirmed by q^1^H NMR spectroscopy and quantified based on the integration of the OC H-1 and H-3 aldehydic proton signals at δ 9.22 and 9.63, respectively, relative to the residual CHCl_3_ peak in CDCl_3_ at δ 7.24 [37].

### 2.2. Cell Line and Culture Conditions

The human CRC cell line HCT-116 was obtained from Charles River Laboratories (Wilmington, MA, USA). Cells were cultured in RPMI-1640 medium supplemented with 10% fetal bovine serum (FBS), 100 U/mL penicillin G, and 100 μg/mL streptomycin, and maintained at 37 °C in a humidified incubator with 5% CO_2_. For routine passaging, cells were washed with Ca^2+^/Mg^2+^-free phosphate-buffered saline (PBS) and detached using 0.05% trypsin containing 0.02% EDTA for 3–5 min at 37 °C before subculturing.

### 2.3. Experimental Treatments

A 25 mM stock solution of OC was prepared in sterile dimethyl sulfoxide (DMSO) and diluted to the desired concentrations for individual experiments. The final DMSO concentration was kept constant across all treatment groups and did not exceed 0.1% (*v*/*v*). To preserve compound stability, OC was stored under N_2_ in amber, airtight glass vials at −20 °C, protected from light and moisture, and thawed only once immediately before use [8,33,34,37].

### 2.4. Western Blot Analysis

Primary tumor tissues were collected immediately after animal sacrifice, snap-frozen, and stored at −80 °C until protein extraction. Tumors were homogenized in RIPA lysis buffer, and protein concentrations were determined using the Pierce BCA Protein Assay (Thermo Fisher Scientific, Rockford, IL, USA). Equal amounts of protein were separated on 10% SDS-PAGE gels and transferred onto polyvinylidene difluoride (PVDF) membranes. Membranes were blocked with an EveryBlot Blocking Buffer (Bio-Rad, Hercules, CA, USA) before incubation with the appropriate primary antibodies, followed by horseradish peroxidase-conjugated secondary antibodies. Protein bands were visualized using the ChemiDoc XRS imaging system and quantified with Image Lab software v5.2.1 (Bio-Rad). β-tubulin served as the loading control to confirm equal protein loading across samples. All experiments were performed independently three times, and representative blots are displayed in the figures.

### 2.5. Luciferase Labeling of HCT-116 Cells Aided by Lentivirus Transduction

HCT-116 cells were seeded into 12-well plates and allowed to reach approximately 60–70% confluence before lentiviral transduction. Luciferase-expressing lentiviral particles (Kerafast, Boston, MA, USA) were prepared in Opti-MEM reduced-serum medium and added to the cells for 4–6 h. Following transduction, the viral medium was replaced with complete growth medium, and cells were cultured for several days. Stable luciferase-expressing cells were selected and maintained in medium containing 15 μg/mL puromycin (Santa Cruz Biotechnology, Dallas, TX, USA), with fresh selection medium replaced every 2 days. Luciferase expression was confirmed by incubating the cells with a 50 mM XenoLight D-luciferin K^+^ salt substrate (PerkinElmer, Waltham, MA, USA) for 6 min at room temperature, followed by bioluminescence imaging using the IVIS imaging system (PerkinElmer) [8].

### 2.6. Animal Model and Treatment Mode

Male and female athymic nude mice (Foxn1^nu^/Foxn^1+^, 4–5 weeks old) were obtained from Envigo (Indianapolis, IN, USA) and housed in an animal facility at the University of Louisiana Monroe College of Pharmacy [8,31,32,33,34,35]. Following a one-week acclimation period, mice were maintained in sterile filter-top cages with autoclaved bedding and provided sterile food and water under controlled environmental conditions (24 ± 2 °C, 50–60% relative humidity, and a 12 h light/dark cycle). Cages were changed twice weekly to maintain optimal hygiene and minimize stress. All animal procedures were approved by the Institutional Animal Care and Use Committee (IACUC) of the University of Louisiana Monroe (Protocols 25-JUL-KES-01 and 25-JUL-KES-02) and conducted in accordance with NIH guidelines. Throughout the study, mice were monitored daily for body weight, food and water intake, general health, and signs of distress to ensure animal welfare. Upon study completion, anesthetized animals were euthanized using cervical dislocation according to AVMA Guidelines on Euthanasia from 2020, https://www.avma.org/resources-tools/avma-policies/avma-guidelines-euthanasia-animals, accessed on 6 August 2026.

### 2.7. Orthotopic CRC Xenograft Model

Orthotopic CRC was established in each isoflurane-anesthetized mouse before subjected to a 1 cm midline incision in the skin and abdominal wall muscle. The cecum area between the mucosa and the muscularis externa layers of the cecal wall of each nude mouse was carefully exposed. About 5 × 10^6^ HCT-116-Luc cells suspended in 100 μL Matrigel were directly injected into the cecal wall/mucosa of each mouse. The abdominal wall wound was then closed using 25 uL Vetbond (3M Vetbond Tissue Adhesive, Amazon, Seattle, WA, USA), followed by aseptically clipping the sealed wound using Kent reflex clips KC35210 (Kent Scientific, Torrington, CT, USA) [38,39,40]. Each procedure was completed within 5–10 min. Donor animals used for FMT were excluded from xenografting surgery. Bioluminescence tumor engraftment, progression, and metastasis were monitored twice weekly using an IVIS Lumina Series III imaging system (PerkinElmer, Waltham, MA, USA). To investigate the contribution of GM to the anti-tumor activity of OC, a non-systemically absorbable broad-spectrum antibiotics cocktail (ABC) consisting of bacitracin A, neomycin trisulfate, and amphotericin B (Sigma-Aldrich, St. Louis, MO, USA) was administered daily via oral gavage. Each freshly prepared 300 μL dose contained 0.4 mg bacitracin A, 0.4 mg neomycin, and 0.1 mg amphotericin B per mouse. The HCT-116-Luc cell line was selected because it represents *KRAS^G13D^*-mutated CRC with aggressive progression and metastatic profiles and proved sensitive to OC [8]. Once orthotopic tumors were engrafted, guided by bioluminescence imaging, usually within 14–21 days after implantation, mice were randomized into the following four treatment groups (n = 6 males and 6 females per group): (i) oral OC (10 mg/kg/day) administered in 300 μL dephenolized extra-virgin olive oil (dpEVOO); (ii) oral OC (10 mg/kg/day) plus daily oral ABC; (iii) placebo control (300 μL dpEVOO) plus daily oral ABC; and (iv) intraperitoneal OC 10 mg/kg, administered three times weekly. In the combination group ii, ABC was administered 2 h before OC treatment. All daily treatments continued for 21 days. At study completion, mice were anesthetized with isoflurane and euthanized. Primary tumors and major organs were collected, weighed, snap-frozen, and stored at −80 °C for subsequent protein extraction and Western blotting analysis.

### 2.8. Fecal Microbiota Transplantation (FMT) Model

Ten fresh fecal pellets, corresponding to approximately 300–350 mg wet weight, were collected daily between 3:00 PM and 5:00 PM from each gender of healthy donor nude mice that were treated for a week with either daily oral OC at 20 mg/kg or a placebo, dpEVOO. Male and female donor feces were processed and administered separately and were not pooled across sexes, maintaining sex-matched donor-to-recipient transfer throughout the study. The afternoon collection time was intended to minimize circadian variation in GM compositions and afford the highest GM loads. Fecal samples were processed immediately under anaerobic conditions by homogenization in sterile phosphate-buffered saline (PBS; 1:10, *w*/*v*) to yield a final suspension of approximately 100 mg wet feces/mL; these were filtered through sterile 120 μm mesh under nitrogen to remove undigested material and centrifuged to eliminate large debris while enriching the microbial fraction. The total handling time from collection to homogenization was maintained within 30 min to preserve the viability of oxygen-sensitive anaerobic taxa. Freshly prepared 300 µL fecal suspensions were orally administered daily to recipient nude mice immediately after processing. Healthy donor nude mice (*n* = 10; 5 males and 5 females per group) continued daily oral 20 mg/kg OC or dpEVOO treatments throughout the study to maintain the respective donor GM profiles. For the FMT study, recipient mice (18 males and 18 females) were orthotopically implanted with 5 × 10^6^ HCT-116-Luc cells in the cecal wall. To facilitate donor microbiota engraftment, recipient mice received the antibiotics cocktail ABC daily via oral gavage for one week beginning approximately 14 days after tumor xenografting, and before the tumors were established. Mice were then randomized into three groups (*n* = 6 males and 6 females per group): (i) daily oral OC (10 mg/kg in dpEVOO); (ii) daily FMT from OC-treated donor mice; or (iii) daily FMT from placebo control-treated donor mice. Treatments continued daily for 21 days. Mice were anesthetized with isoflurane and euthanized at the end of the study. Primary tumors and major organs were collected, weighed, snap-frozen, and stored at −80 °C for subsequent protein extraction and Western blot analysis. FMT was administered by oral gavage, which was preferred over rectal enema because it is less invasive, avoids the risk of rectal injury, and does not require repeated sedation. Healthy nude mice were used as donors based on their robust GM profile diversity [36].

### 2.9. Statistics

Statistical analyses were performed using GraphPad Prism version 8.4.3 (GraphPad Software, La Jolla, CA, USA). Data are presented as mean ± standard deviation (SD). Comparisons between groups were analyzed using one-way ANOVA, and repeated-measures two-way ANOVA was used to assess mice’s body weight. Statistical significance was defined as * *p* < 0.05, ** *p* < 0.01, *** *p* < 0.001 and **** *p* < 0.0001.

## 3. Results

### 3.1. Comparison of Oral and Intraperitoneal OC Suppressive Efficacy Against CRC Progression in the Presence and Absence of Broad-Spectrum Antibiotics Cocktail ABC in Male and Female Nude Mice

**Figure 1:**
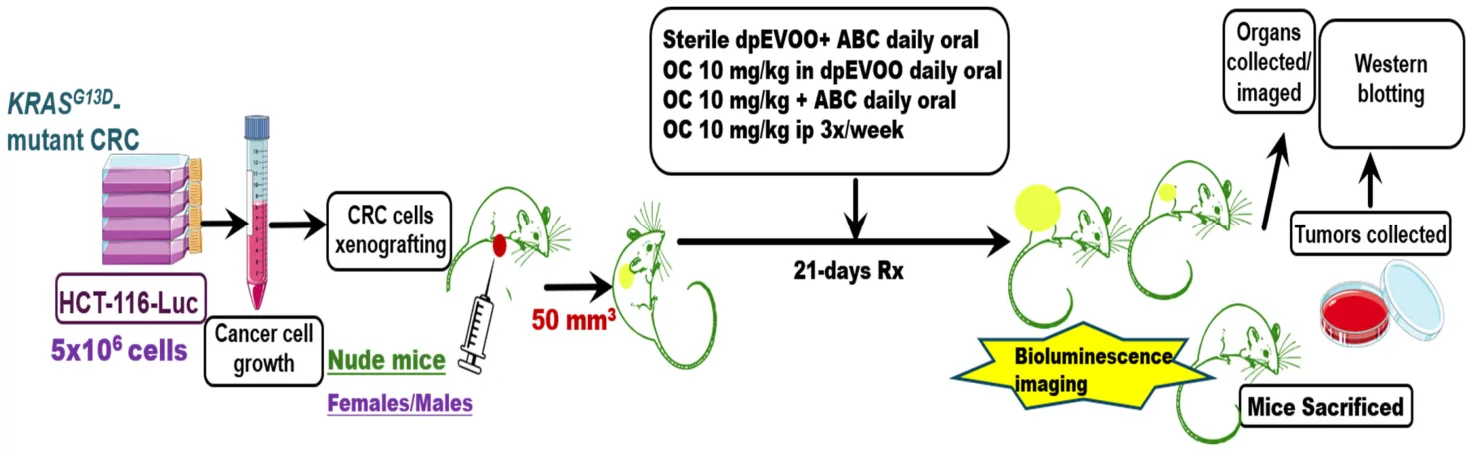
Schematic overview of the orthotopic intra-cecally xenografted HCT-116-Luc colorectal cancer (CRC) nude mouse model and experimental design used to assess the contribution of gut microbiota (GM) to the anti-CRC activity of oleocanthal (OC), comparing oral OC treatment in the presence or absence of antibiotic cocktail (ABC) with placebo control and intraperitoneal OC treatment.

**Figure 2:**
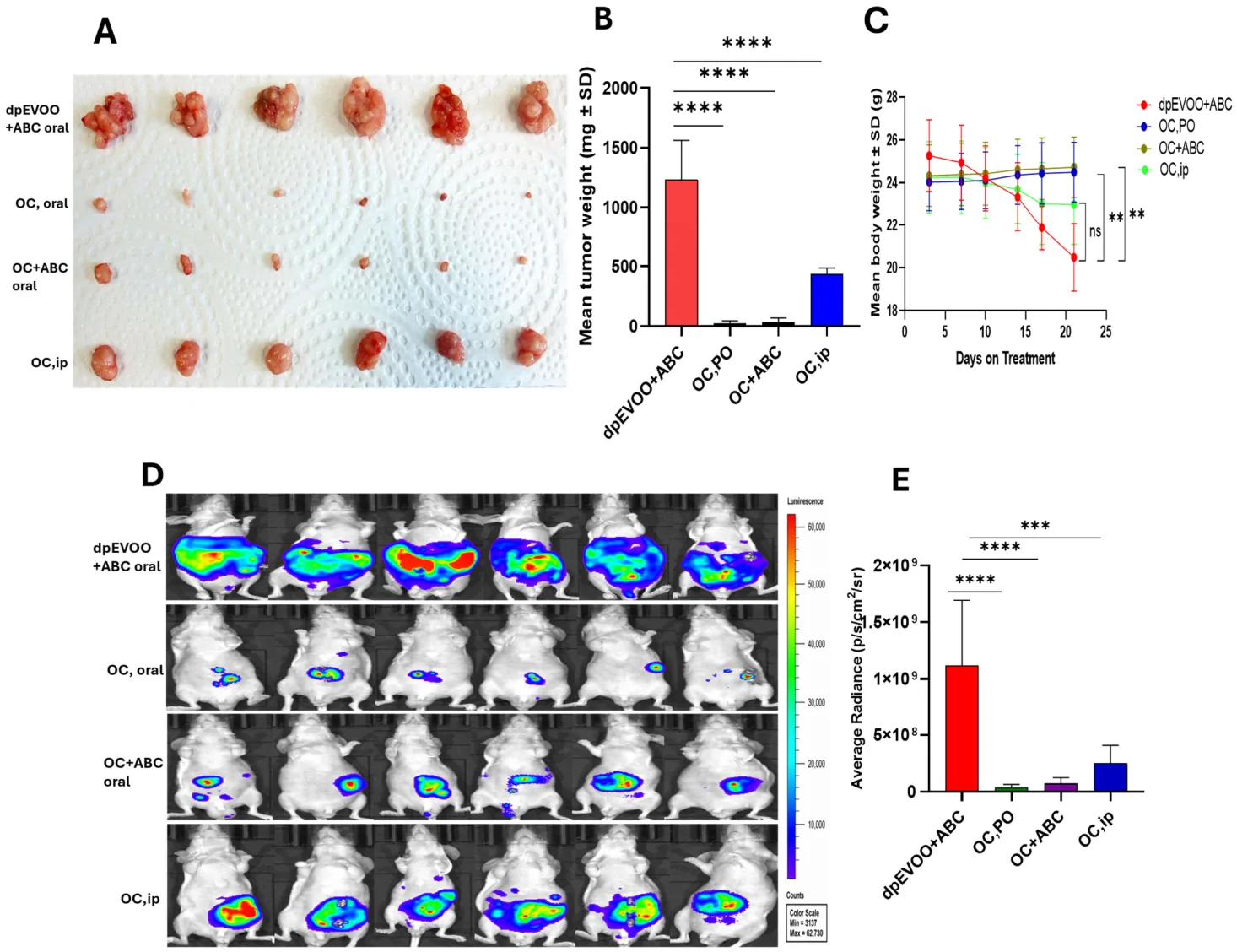
**Oleocanthal** (OC) anti-colorectal cancer (CRC) activity in orthotopically intra-cecally xenografted male nude mice. Oral OC showed greater suppression of orthotopic HCT-116-Luc tumor progression compared with intraperitoneal OC administration in male nude mice. (**A**) Representative images of excised primary tumors from mice treated with 10 mg/kg oral OC in dephenolized extra-virgin olive oil with antibiotics cocktail (dpEVOO + ABC), oral OC (10 mg/kg; oral), oral OC plus antibiotics (OC + ABC), or intraperitoneal OC (10 mg/kg, ip). (**B**) Mean tumor weight (mg ± SD) at study endpoint. (**C**) Mean body weight (g ± SD) monitored throughout the treatment period. (**D**) Bioluminescence images of intra-cecally xenografted HCT-116-Luc tumor-bearing male nude mice at study end. (**E**) Quantitative monitoring of tumor bioluminescence intensity in live animals. Data presented as mean ± SD (n = 6 mice/group). ** *p* < 0.01, *** *p* < 0.001, **** *p* < 0.0001, ns indicates no statistical significance.

To determine the contribution of GM to the anti-CRC efficacy of OC, four groups of male nude mice bearing orthotopically intra-cecally xenografted HCT-116-Luc tumors were treated as follows: 1. sterile placebo dpEVOO + ABC, daily oral; 2. OC in dpEVOO, 10 mg/kg, daily oral; 3. OC 10 mg/kg combined with non-systemically absorbable broad-spectrum ABC, daily oral; and 4. OC 10 mg/kg in sterile dpEVOO, intraperitoneal (ip), 3×/week (Figure 1). Gross examination of excised tumors after three dosing weeks revealed marked suppression of tumor progression in oral OC and OC + ABC groups, compared with the dpEVOO placebo control + ABC group. By contrast, mice treated with the OC ip route exhibited intermediate tumor growth (Figure 2A).

Quantitative tumor weight analysis assessed various treatment effects (Figure 2B, Table 1 and Supplementary Table S1). The dpEVOO placebo control + ABC group exhibited the highest cumulative tumor burden, with a total tumor weight of 7404.5 mg (Table 1). Oral administration of OC resulted in a profound reduction in tumor burden, yielding a total tumor weight of only 178.4 mg, corresponding to a 97.6% tumor suppression relative to the placebo dpEVOO control group. Similarly, the OC + ABC group showed a total tumor weight of 212.8 mg, representing a 97.1% reduction compared with the placebo control. In contrast, the intraperitoneal OC treatment resulted in a total tumor weight of 2617.7 mg, corresponding to a 64.5% tumor weight reduction relative to the placebo control group; however, this was substantially less effective compared to the OC orally treated group. These findings indicate that oral OC exerted significantly greater anti-CRC activity than ip administration.

Longitudinal body weight monitoring revealed distinct differences among treatment groups (Figure 2C and Supplementary Table S2). Mice in the placebo control + ABC group exhibited a progressive decline in body weight throughout the study, consistent with increased tumor burden and disease progression. A similar trend, although less pronounced, was observed in the OC ip group (Figure 2C). In contrast, body weights remained stable in both oral OC and OC + ABC groups throughout the treatment course, suggesting improved overall health status and notably reduced tumor burden in mice receiving oral OC.

Bioluminescence imaging further corroborated the aforementioned findings (Figure 2D,E and Supplementary Table S3). The mice placebo control + ABC group displayed intense luciferase signals indicative of extensive tumor progression. Oral OC treatment markedly reduced bioluminescent signal intensity, consistent with its pronounced suppression of tumor growth. The OC + ABC group exhibited similarly low signal intensities, whereas intraperitoneal OC treatment resulted in noticeably higher residual tumor-associated bioluminescence. Collectively, these findings demonstrate that oral OC produces substantially greater inhibition of HCT-116-Luc tumor progression than intraperitoneal administration and is associated with preservation of body weight and reduced tumor burden.

**Figure 3:**
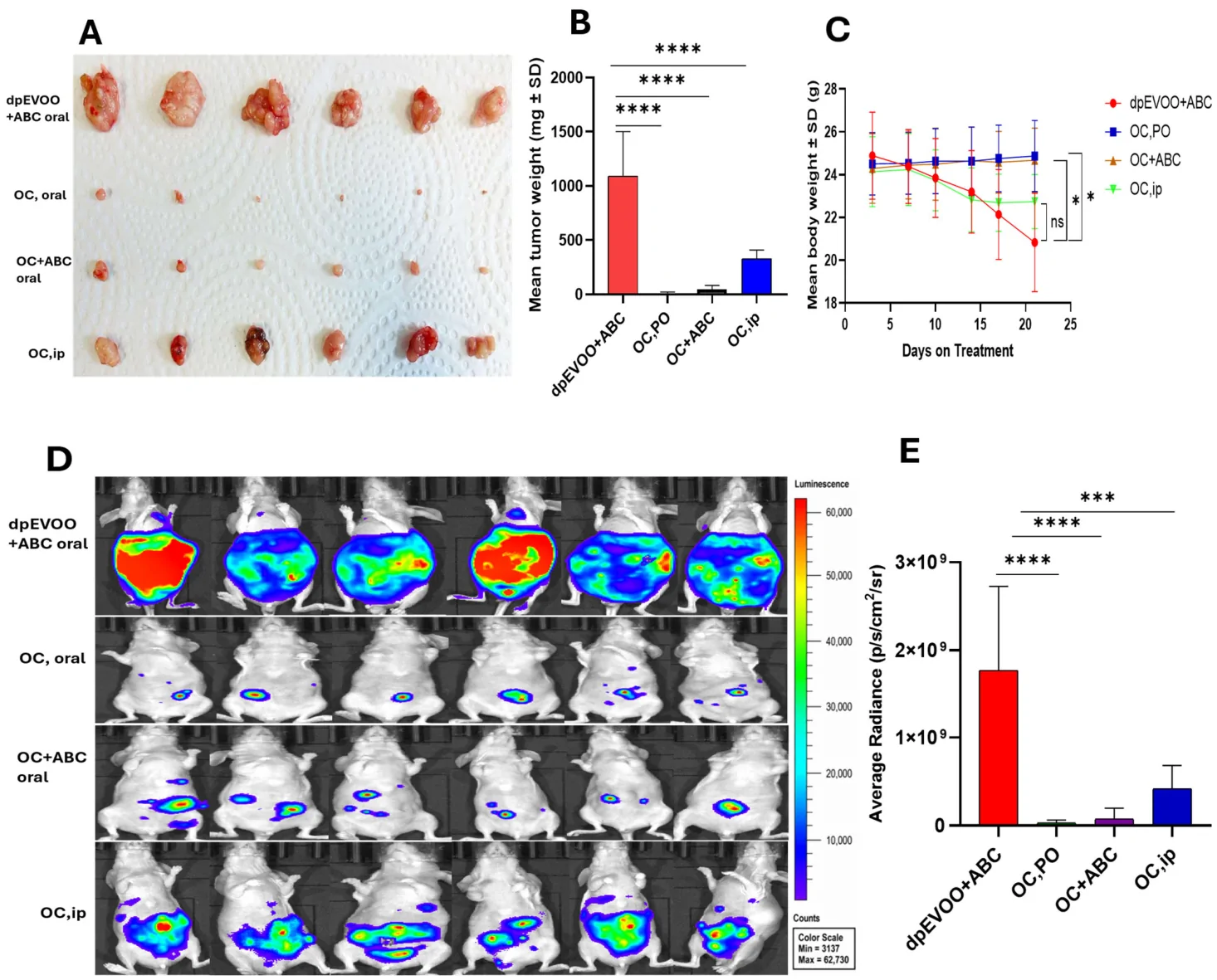
Oral oleocanthal (OC) suppresses HCT-116-Luc tumor progression more effectively than intraperitoneal administration in female nude mice. (**A**) Representative images of excised tumors from mice treated with dephenolized extra-virgin olive oil plus antibiotic cocktail (dpEVOO + ABC), oral (PO) OC (10 mg/kg, oral), oral OC plus antibiotics (OC + ABC), or intraperitoneal OC (10 mg/kg, ip). (**B**) Mean tumor weight (mg ± SD) at study endpoint. (**C**) Mean body weight (g ± SD) during treatment. (**D**) Representative endpoint bioluminescence images of HCT-116-Luc tumors. (**E**) Tumor bioluminescence intensity quantitative monitoring in mice. Data presented as mean ± SD (n = 6 mice/group). * *p* < 0.05, *** *p* < 0.001, **** *p* < 0.0001, ns indicates no statistical significance.

To evaluate the gender differences in GM’s contribution to OC anti-CRC, the same experiment described earlier in male nude mice was repeated with the female mice following the same four group treatments. A gross examination of excised primary tumors revealed substantial differences in tumor burden among treatment groups (Figure 3A). Similar to the male mice results, female mice receiving oral OC exhibited the smallest tumors, whereas the placebo control dpEVOO + ABC group developed large tumor masses. Although OC + ABC treatment reduced tumor growth relative to the control group, tumors were visibly larger than those observed in mice receiving oral OC alone. Intraperitoneal OC treatment also produced intermediate tumor suppression.

Tumor weight analysis assessed the treatment effect for each group (Figure 3B and Table 1). The placebo control + ABC group exhibited the highest cumulative tumor burden, with a total tumor weight of 6540.1 mg (Table 1). Oral OC treatment markedly suppressed tumor progression, resulting in a total tumor weight of 62.6 mg, corresponding to a 99.1% tumor reduction relative to the placebo control group. In contrast, mice treated with OC + ABC exhibited a total tumor weight of 262.6 mg, representing a 96.0% tumor reduction compared with the placebo control. Intraperitoneal administration of OC reduced the total tumor weight to 1968.0 mg, corresponding to a 69.9% reduction compared to the control group; however, the tumor burden remained substantially greater than that observed following OC oral administration.

Similar to the male mice experiment, body weight monitoring demonstrated a progressive decline in the placebo control + ABC group throughout the treatment period, consistent with increased tumor burden and disease progression (Figure 3C). A similar trend was observed in the OC ip group during the later stages of the study. In contrast, body weights remained relatively stable in both the OC oral and OC + ABC oral groups, indicating improved tolerability and controlled disease-associated weight loss.

Bioluminescence imaging further corroborated the tumor weight findings (Figure 3D,E). Mice in the placebo control + ABC group displayed intense bioluminescence signals, indicative of extensive tumor progression. Oral OC treatment produced the greatest reduction in bioluminescent signal intensity, whereas ABC-mediated GM depletion partially diminished this response, as evidenced by the increased signal intensity observed in the OC + ABC group. Likewise, intraperitoneal OC treatments resulted in substantially higher residual bioluminescent signals than oral OC administration, further suggesting the benefits of the oral route for OC-improved anti-CRC activity.

### 3.2. Oral OC-Suppressed Multi-Organ Metastatic Dissemination More Effectively than Intraperitoneal Administration in Both Male and Female Nude Mice

**Figure 4:**
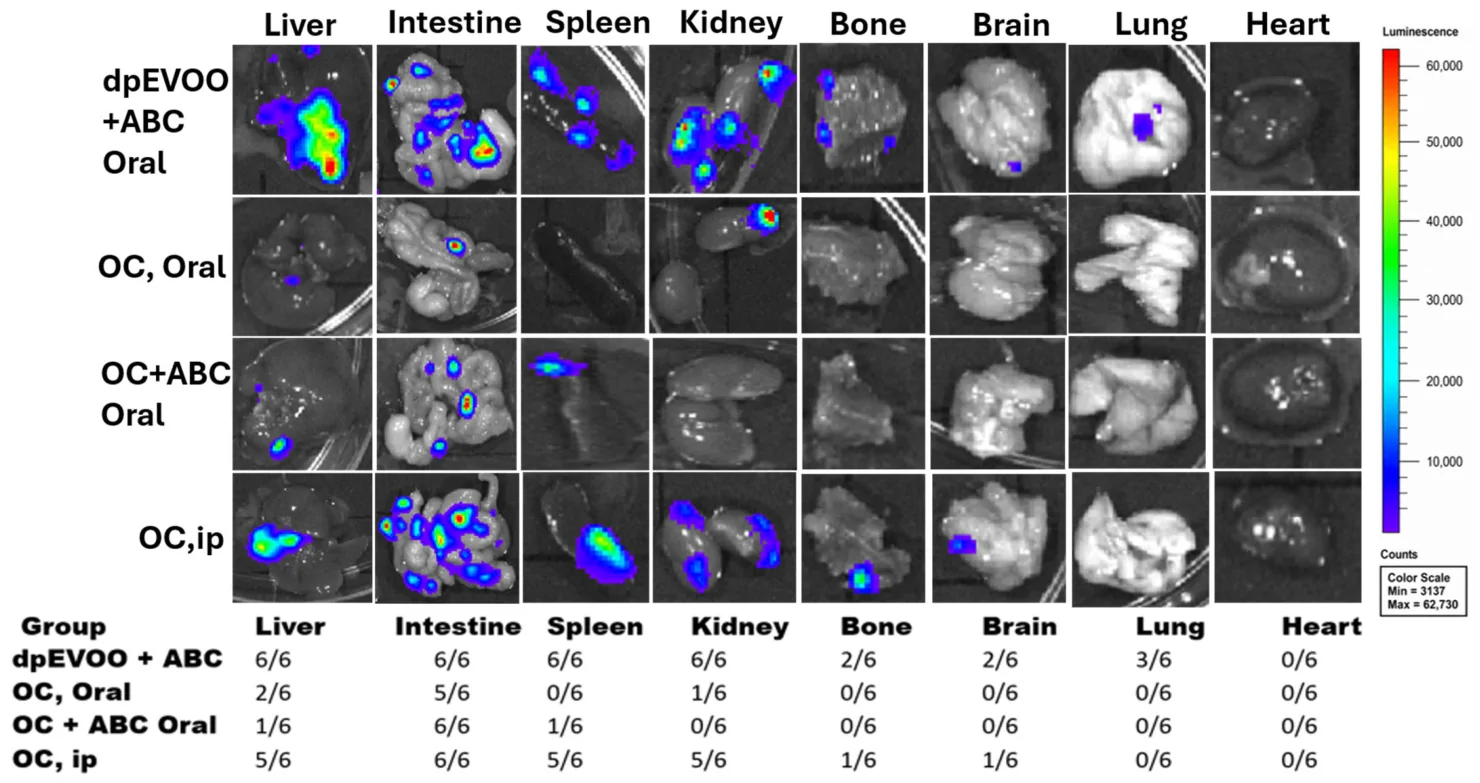
Representative bioluminescence images and quantitative analysis of HCT-116-Luc colorectal cancer (CRC) metastatic dissemination in major male nude mouse organs.

The impact of OC treatments on metastatic dissemination of the *KRAS^G13D^*-mutant CRC HCT-116-Luc tumors was assessed at the study’s termination using luciferin-aided bioluminescence imaging of eight collected animal organs, including the liver, intestine, spleen, kidney, bone, brain, lung, and heart (Figure 4, Supplementary Figure S1, and Supplementary Table S3). In male mice (Figure 4), the placebo control + ABC group exhibited an extensive metastatic spread, with 100% incidence in the liver, intestine, spleen, and kidney, accompanied by metastases to the bone (2/6), brain (2/6), and lung (3/6). Oral OC markedly reduced metastatic burden, limiting liver metastasis to 2/6 mice and completely preventing metastasis to the spleen, bone, brain, lung, and heart. Intestinal involvement remained the predominant metastatic site (5/6). Similarly, the OC + ABC group demonstrated reduced metastatic dissemination, with liver and spleen metastases observed in only 1/6 mice and complete absence of metastasis in the kidney, bone, brain, lung, and heart. In contrast, intraperitoneal OC provided substantially less protection, with persistent metastatic involvement of the liver (5/6), intestine (6/6), spleen (5/6), and kidney (5/6), indicating inferior efficacy of the OC intraperitoneal route in suppressing systemic CRC dissemination (Figure 4).

**Figure 5:**
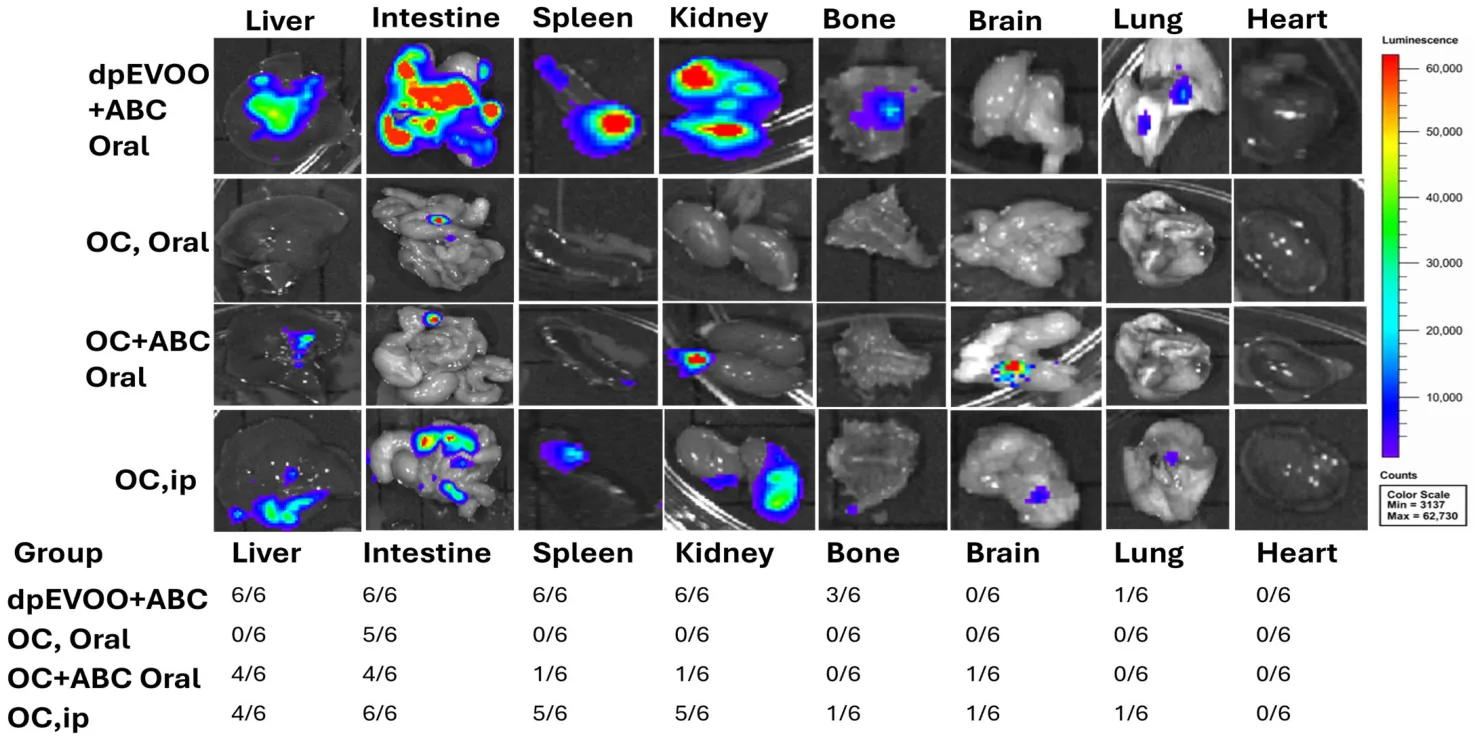
Representative bioluminescence images and quantitative analysis of HCT-116-Luc colorectal cancer (CRC) metastatic dissemination in major female nude mouse organs.

A similar pattern was observed in female mice (Figure 5 and Supplementary Figure S1). The dpEVOO + ABC control group displayed widespread metastatic involvement, including the liver, intestine, spleen, and kidney in all animals, with additional metastases detected in the bone (3/6) and lung (2/6) (Figure 5). Oral OC provided the greatest protection against metastatic progression, completely preventing metastasis to the liver, spleen, kidney, bone, brain, lung, and heart, while reducing intestinal involvement to 5/6 mice. ABC-mediated GMdepletion partially diminished this protective effect, as evidenced by increased metastatic incidence in the liver (4/6), intestine (4/6), spleen (1/6), kidney (1/6), and brain (1/6) in the OC + ABC group. Consistent with the findings in male mice, intraperitoneal OC treatments were associated with extensive metastatic dissemination, including involvement of the liver (4/6), intestine (6/6), spleen (5/6), and kidney (5/6), with occasional metastases to the bone, brain, and lung (Figure 5).

Collectively, these findings demonstrate that oral OC markedly suppresses multi-organ metastatic dissemination of HCT-116-Luc tumors in both male and female nude mice. The reduced antimetastatic efficacy observed following ABC-mediated GM depletion, particularly in female mice, suggests that GM contributes to the oral OC protective antimetastatic effects. Furthermore, the substantially greater metastatic burden observed following intraperitoneal administration further validates the contribution of GM to OC anti-CRC progression and antimetastatic effects. Analogous to progression-suppressive efficacy, OC showed numerically greater antimetastatic response in female versus male nude mice, as evidenced by absolute success in preventing HCT-116-Luc metastasis to female mouse livers (Figure 5).

### 3.3. OC Suppressed SMYD2 and EZH2 Expressions in HCT-116 Collected Tumors

**Figure 6:**
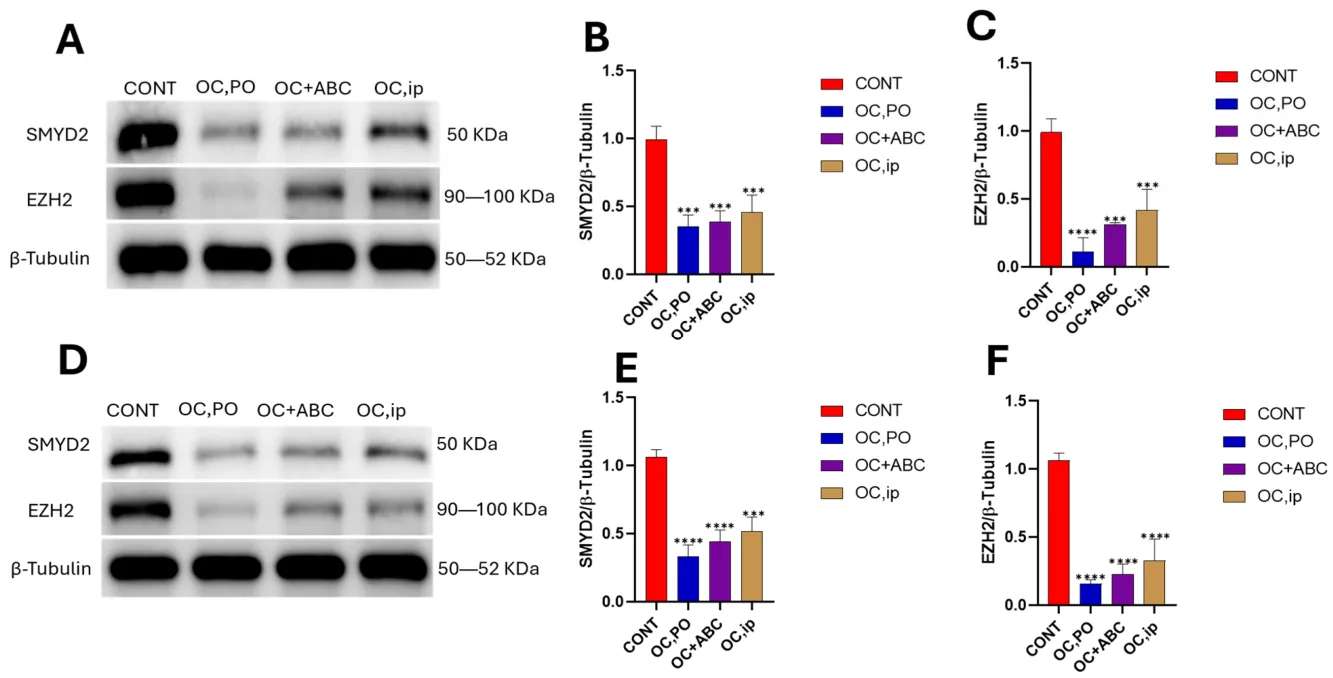
Western blot analysis of SMYD2 and EZH2 expression in HCT-116-Luc primary tumors. Tumor lysates were prepared by pooling equal amounts of primary tumor tissues from each treatment group within each mouse gender (*n* = 6) prior to protein extraction and analysis. (**A**–**C**) Male mice; (**D**–**F**) female mice. (**A**,**D**) Representative blots of SMYD2, EZH2, and β-tubulin (loading control) in placebo control (CONT), oral (PO) oleocanthal (OC), oral oleocanthal plus antibiotics cocktail (OC + ABC), and OC intraperitoneal (ip) groups. (**B**,**E**) SMYD2 and (**C**,**F**) EZH2 quantification normalized to β-tubulin. Data presented as mean ± SEM relative to control group (CONT). *** *p* < 0.001, **** *p* < 0.0001 vs. CONT (one-way ANOVA).

The epigenetic drivers lysine methyltransferases SMYD2 and EZH2 have been reported as prospective OC molecular targets in *KRAS^G13D^*-mutant CRC HCT-116 cells [8]. To investigate the epigenetic basis underlying the GM-associated anti-CRC activity of OC, we evaluated the expression levels of SMYD2 and EZH2 in collected primary HCT-116-Luc tumors by Western blot analysis. Tumor lysates from the dpEVOO + ABC control, OC oral 10 mg/kg, OC + ABC, and OC ip 10 mg/kg, 3×/week groups were probed for SMYD2 and EZH2, with β-tubulin serving as the loading control in male- and female-treated mouse groups (Figure 6 and Supplementary Figure S2). In male mice (Figure 6A–C and Supplementary Table S4), SMYD2 expression was significantly reduced in all three OC-treated groups relative to placebo control group (CONT), with the oral OC group showing the most pronounced suppression (~65% reduction; *p* < 0.001), followed closely by OC + ABC (~62% reduction; *p* < 0.001) and finally the OC ip group (~54% reduction; *p* < 0.001). A similar trend was observed for EZH2, where oral OC produced the greatest suppression (~88% reduction; *p* < 0.0001), followed by OC + ABC (~70% reduction; *p* < 0.001) and OC ip (~57% reduction; *p* < 0.001) relative to the placebo control dpEVOO.

In female mice (Figure 6D–F), the pattern was consistent with male animals. SMYD2 expression was reduced by approximately 71% in the oral OC group (*p* < 0.0001), 56% in the OC + ABC group (*p* < 0.0001), and 52% in the OC ip group (*p* < 0.001) relative to placebo control. EZH2 suppression followed the same hierarchy, with the oral OC group exhibiting the most robust downregulation (86% reduction; *p* < 0.0001), followed by OC + ABC (77% reduction; *p* < 0.0001), and finally, the OC ip group (~68% reduction; *p* < 0.0001). Across both sexes, the oral OC group consistently exhibited the greatest suppression of SMYD2 and EZH2 expression, while the OC ip group showed the least suppression among the three treatment groups. The OC + ABC group displayed an intermediate phenotype, partially attenuating the epigenetic suppressive effect of oral OC relative to the oral OC group alone.

### 3.4. Effect of FMT on KRAS-Mutant CRC Progression in Male and Female Nude Mice

**Figure 7:**
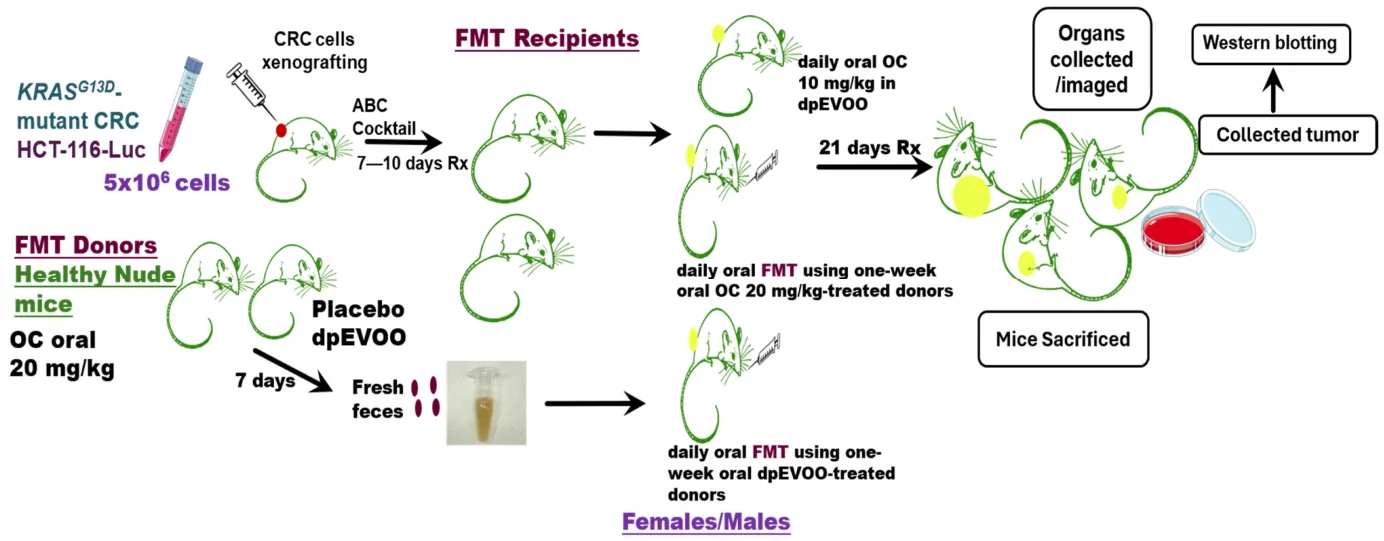
Schematic illustration of the fecal microbiota transplantation (FMT) experimental design used to evaluate direct anti-colorectal cancer (CRC) activity of oleocanthal (OC)-modulated gut microbiota (GM).

To determine whether GM modulated by oral OC directly contributes to its anti-CRC activity, fecal microbiota transplantation (FMT) was performed using fresh fecal material collected from high OC dose (20 mg/kg daily oral)-treated donor mice. Recipient male nude mice bearing HCT-116-Luc tumors received FMT from either placebo dpEVOO-treated donors (FMT control) or OC 20 mg/kg treated donors (FMT treatment) for 21 days, after which the therapeutic outcomes were compared with direct OC 10 mg/kg daily oral administration (OC, oral) (Figure 7).

**Figure 8:**
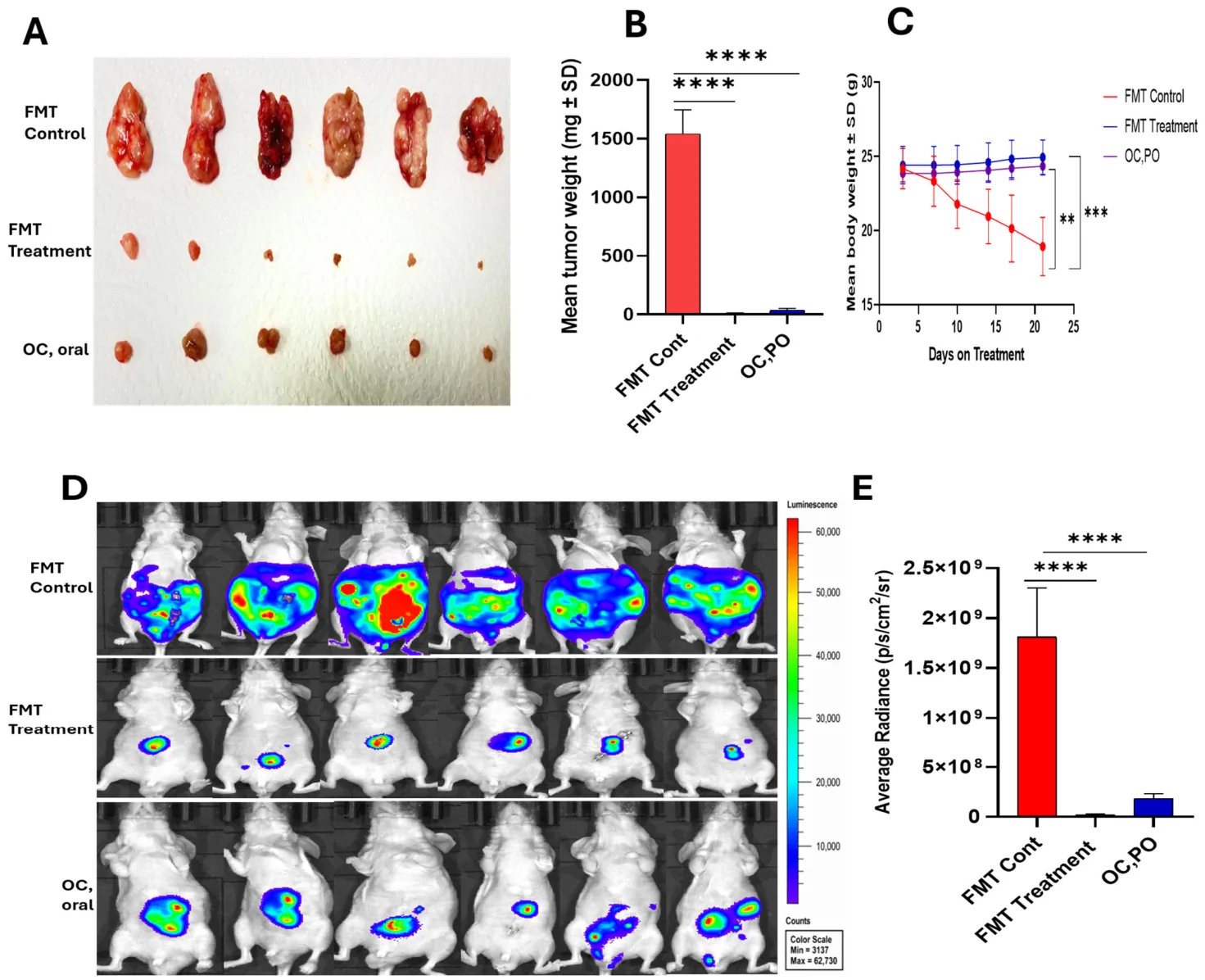
Anti-colorectal cancer (CRC) activity of fecal microbiota transplantation (FMT) treatments from oleocanthal (OC)-treated donors against HCT-116-Luc in male nude mice. (**A**) Representative images of excised tumors from male mice receiving FMT from vehicle-treated donors (FMT control), FMT from OC-treated donors (FMT treatment), or oral (PO) 10 mg/kg OC. (**B**) Mean tumor weight (mg ± SD) at the study’s endpoint. (**C**) Mean body weight (g ± SD) monitored throughout the treatment period. (**D**) Representative endpoint bioluminescence images of HCT-116-Luc tumors. FMT from OC-treated donors markedly reduced tumor burden and bioluminescent signal intensity, comparable to direct oral OC treatment, while maintaining stable body weight. (**E**) Quantitative monitoring of tumor bioluminescence intensity in male mice. Data presented as mean ± SD (*n* = 6 mice/group). ** *p* < 0.01, *** *p* < 0.001, **** *p* < 0.0001.

Gross examination of excised tumors in male nude mice at the study’s termination revealed a marked reduction in tumor size in both the FMT treatment and oral OC groups relative to the FMT control group (Figure 8A). Mice receiving FMT from OC-treated donors developed only small residual tumors, closely resembling those observed in mice treated directly with oral OC.

Tumor weight analysis assessed treatment outcomes (Figure 8B and Table 2). The FMT control group exhibited the highest cumulative tumor burden, with a total tumor weight of 9252.2 mg. In contrast, mice receiving FMT from OC-treated donors showed a dramatic reduction in tumor burden, with a total tumor weight of only 48.5 mg, representing a reduction of 99.5% relative to the FMT control group. Similarly, oral OC treatment reduced the total tumor weight to 211.2 mg, corresponding to a 97.7% reduction compared with controls. Notably, FMT from OC-treated donors produced an even lower overall tumor burden than direct oral OC administration.

Longitudinal monitoring identified progressive body weight loss in the FMT control group throughout the study period, consistent with advanced tumor progression. In contrast, body weights remained stable in both the FMT treatment and oral OC groups, indicating reduced disease burden and improved overall health status (Figure 8C).

Bioluminescence imaging further corroborated the gross and quantitative tumor findings (Figure 8D,E and Supplementary Table S5). Mice receiving FMT from vehicle-treated donors exhibited intense luciferase signals indicative of extensive tumor progression. Conversely, both FMT from OC-treated donors and direct oral OC administration markedly suppressed tumor-associated bioluminescence, reflecting substantial inhibition of tumor growth. These findings demonstrate that transplantation of GM from OC-treated donors is sufficient to reproduce the potent anti-CRC effects of oral OC in HCT-116-Luc tumor-bearing mice.

A similar FMT study was also performed in female nude mice with orthotopically xenografted HCT-116-Luc tumors. Female mice received FMT from either placebo dpEVOO-treated female donors (FMT control) or OC-treated donors (FMT treatment), and the therapeutic outcomes were compared with direct daily oral 10 mg/kg OC administration over 21 days.

**Figure 9:**
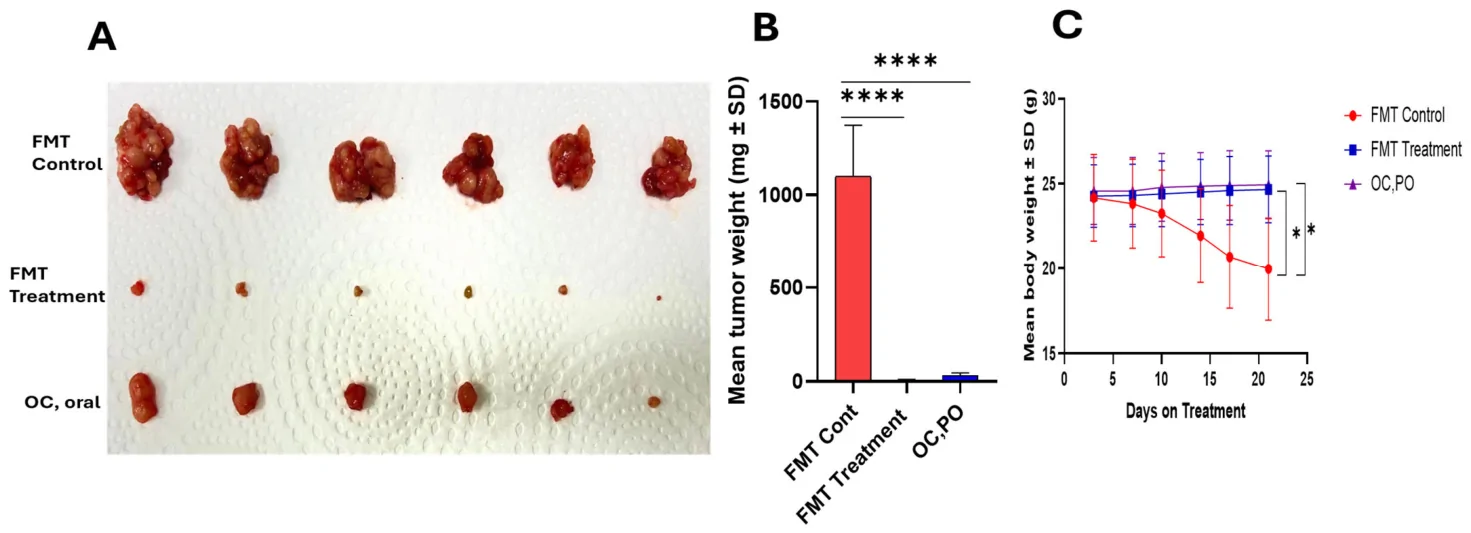

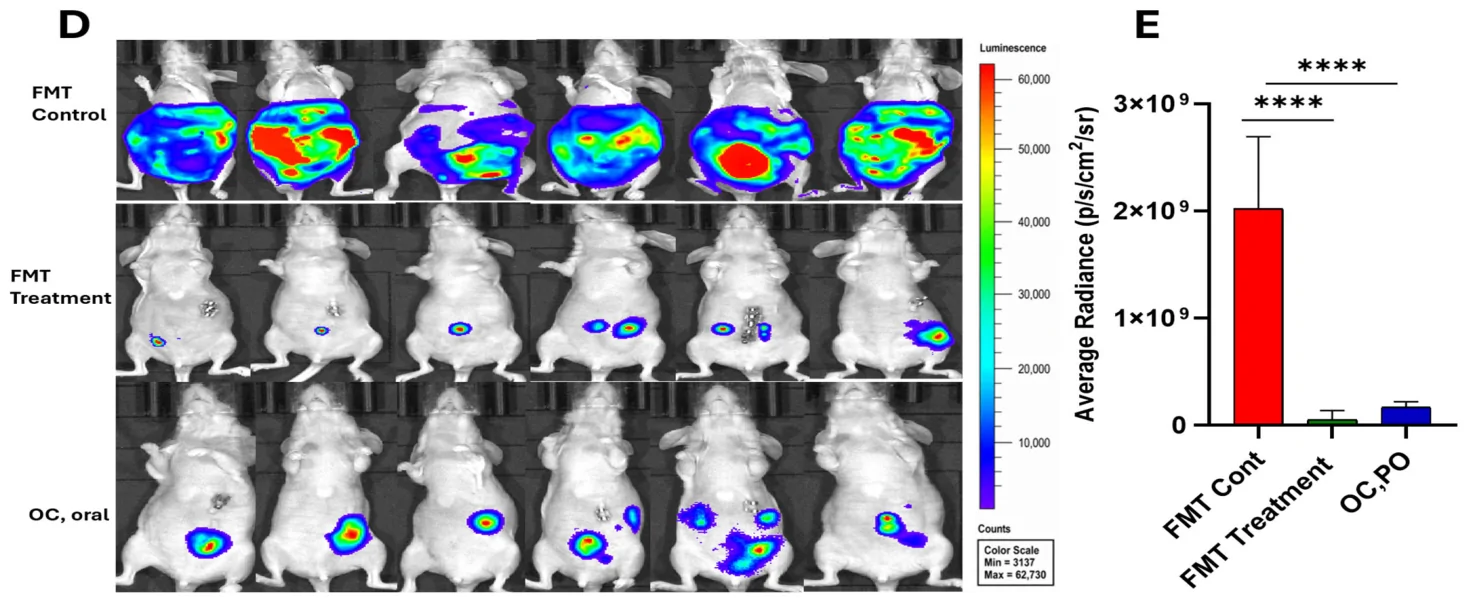
Anti-CRC activity of FMT treatments from OC-treated donors against HCT-116-Luc in female nude mice. (**A**) Representative images of excised tumors from mice receiving FMT from placebo-treated donors (FMT Cont), FMT from OC-treated donors (FMT treatment), or daily oral (PO) OC 10 mg/kg. (**B**) Mean tumor weight (mg ± SD) at the study endpoint. (**C**) Mean body weight (g ± SD) monitored throughout the treatment period. (**D**) Representative endpoint bioluminescence images of HCT-116-Luc tumors. FMT from OC-treated donors markedly reduced tumor burden and bioluminescent signal intensity, comparable to direct oral OC treatment, while maintaining stable body weight. (**E**) Quantitative monitoring of tumor bioluminescence intensity in female mice. Data presented as mean ± SD (n = 6 mice/group). * *p* < 0.05, **** *p* < 0.0001.

Gross examination of excised tumors at the study’s termination demonstrated a substantial reduction in tumor burden in both the FMT treatment and OC oral groups compared with the FMT control group (Figure 9A). Mice receiving FMT from OC-treated donors developed only small residual tumors, comparable to those observed following direct oral OC treatment.

The FMT control group exhibited the highest cumulative tumor burden, with a total tumor weight of 6594.8 mg (Figure 9B and Table 2). In contrast, FMT from OC-treated donors reduced the total tumor weight to 38.4 mg, corresponding to a 99.4% reduction relative to the FMT control group. Similarly, oral OC treatment reduced the total tumor weight to 186.8 mg, representing a 97.2% reduction compared with the controls. Notably, the FMT female mice treatment group exhibited the lowest overall tumor burden among all treatment groups, consistent with previous progression and metastasis findings.

Body weight monitoring revealed a progressive decline in the FMT control group throughout the study period, consistent with advanced tumor progression and increased disease burden (Figure 9C). In contrast, body weights remained stable in both the FMT treatment and oral OC groups, indicating improved overall health status and reduced tumor-associated weight loss.

Bioluminescence imaging further corroborated the gross and quantitative tumor findings (Figure 9D,E). Female mice in the FMT control group displayed intense luciferase signals indicative of extensive tumor progression, whereas mice receiving FMT from OC-treated donors exhibited markedly reduced bioluminescent signal intensity. A similar reduction was observed in the oral OC group, confirming substantial inhibition of tumor progression. These findings demonstrate that FMT from OC high-dose-treated donors effectively recapitulates the anti-CRC activity of oral OC in female HCT-116-Luc tumor-bearing mice.

### 3.5. FMT Suppressed Multi-Organ Metastatic Dissemination in Both Male and Female Nude Mice

**Figure 10:**
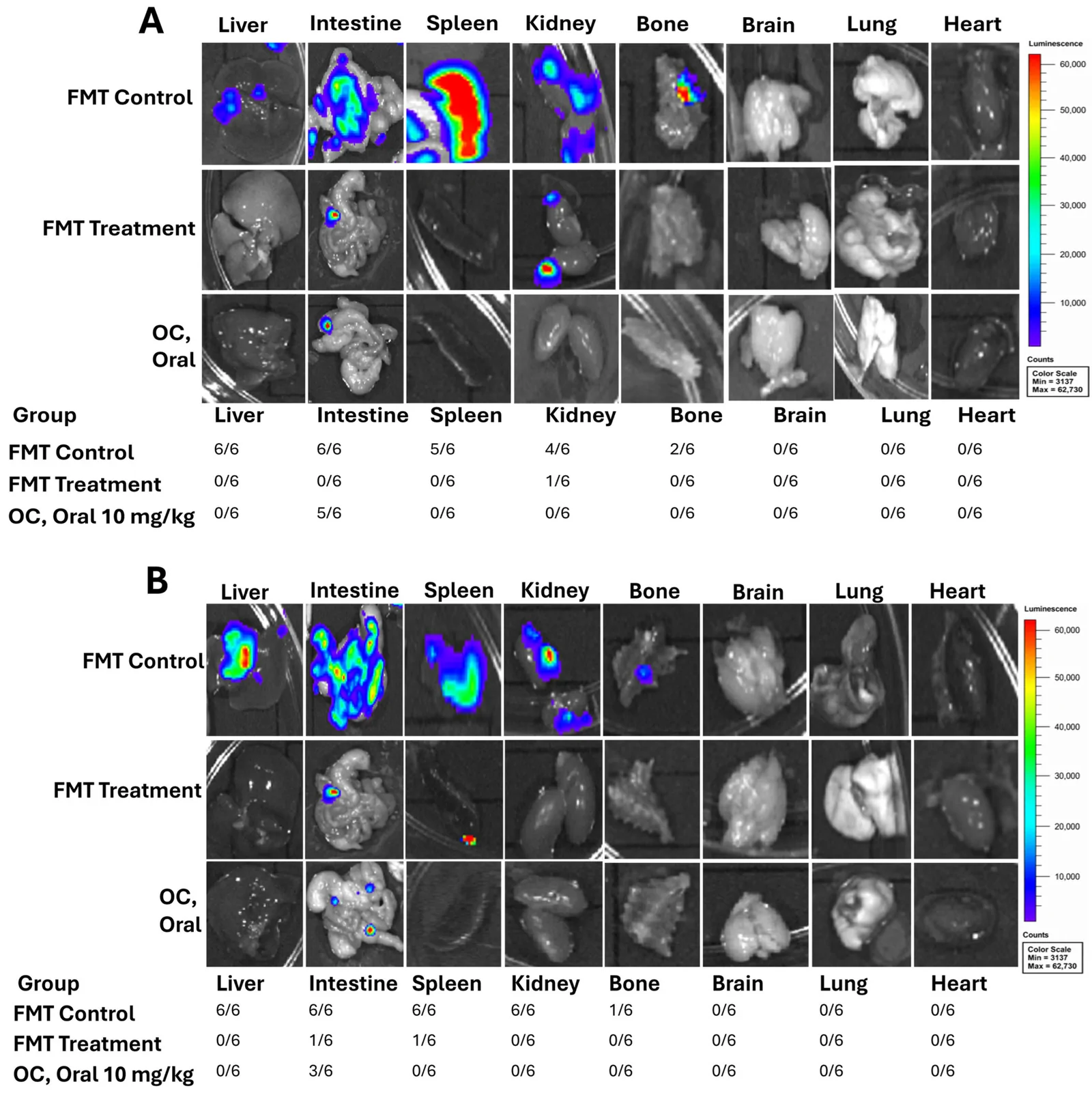
Representative bioluminescence images and quantitative analysis of metastatic dissemination in major isolated organs from (**A**) male and (**B**) female nude mice with orthotopic HCT-116-Luc colorectal cancer (CRC) following the fecal microbiota transplantation (FMT) study.

To determine whether the antimetastatic activity of oral OC could be transferred through FMT, metastatic dissemination in isolated animal organs was evaluated at the study’s end in recipient mice following the FMT experiment. Metastatic involvement was assessed across eight organs, including the liver, intestine, spleen, kidney, bone, brain, lung, and heart (Figure 10A,B and Supplementary Figure S3).

In male mice (Figure 10A), the FMT control group exhibited extensive metastatic dissemination, showing bioluminescent metastatic lesions in the liver (6/6), intestine (6/6), spleen (5/6), and kidney (4/6), together with occasional bone metastases (2/6). In contrast, male mice receiving FMT from OC-treated donors demonstrated near-complete suppression of metastatic spread. Metastatic involvement was limited to a single case of kidney metastasis (1/6), while no metastatic lesions were detected in the liver, intestine, spleen, bone, brain, lung, or heart. Similarly, oral OC treatment completely prevented metastatic dissemination to all organs except the intestine, where minor metastases were observed in 5/6 mice.

A comparable pattern was observed in female mice (Figure 10B). The FMT control group displayed widespread metastatic involvement, with metastases detected in the liver, intestine, spleen, and kidney of all animals (6/6 each), as well as occasional bone involvement (1/6). In a striking contrast, FMT from OC-treated female mouse donors almost completely abrogated metastatic dissemination, with only minor intestinal metastasis (1/6) and spleen metastasis (1/6) detected. Oral OC treatment also markedly reduced metastatic burden, limiting metastatic spread exclusively to the intestine (3/6) while completely preventing metastasis to all other organs examined.

These findings demonstrate that FMT from high-dose OC-treated donors confers potent protection against metastatic dissemination in both male and female HCT-116-Luc CRC-bearing mice. Notably, FMT treatment exhibited antimetastatic efficacy comparable to, and in some organs exceeding, that observed with direct oral OC administration.

### 3.6. FMT Suppressed SMYD2 and EZH2 Protein Expression in Recipient KRAS-Mutant CRC in Both Male and Female Nude Mice

**Figure 11:**
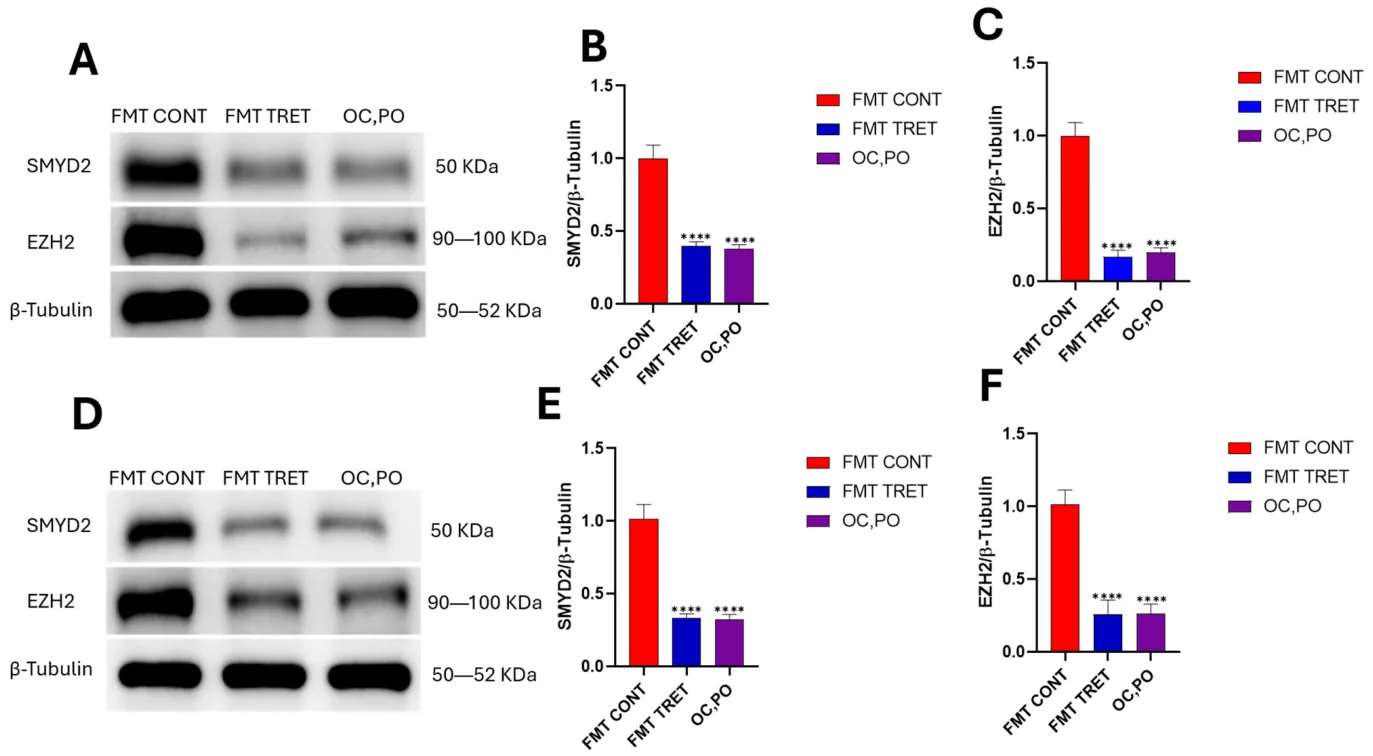
Western blot analysis of SMYD2 and EZH2 expression levels in HCT-116-Luc primary tumors following the fecal microbiota transplantation (FMT) study. Primary tumor tissues from all mice within each treatment group (*n* = 6) were pooled in equal amounts to prepare tumor lysates for protein extraction and analysis. (**A**–**C**) Male mice; (**D**–**F**) female mice. (**A**,**D**) Representative blots of SMYD2 (50 kDa), EZH2 (90–100 kDa), and β-tubulin (loading control) in FMT control (FMT CONT), FMT treatment (FMT TRET), and oral (PO) OC groups. (**B**,**E**) SMYD2 and (**C**,**F**) EZH2 quantification normalized to β-tubulin. Data presented as mean ± SEM relative to FMT CONT. **** *p* < 0.0001 versus FMT CONT (one-way ANOVA).

To determine whether the anti-CRC efficacy of FMT is associated with epigenetic reprogramming, protein expression of SMYD2 and EZH2 was assessed by Western blot in primary tumor lysates from FMT controls, FMT treatments, and oral OC 10 mg/kg treated mice, with β-tubulin used as the loading control (Figure 11A,D).

In male mice (Figure 11A–C), SMYD2 expression was significantly reduced in both the FMT treatment and oral OC groups relative to the FMT control, with each group exhibiting an approximately 60–62% reduction in normalized SMYD2 expression (*p* < 0.0001). EZH2 expression was similarly suppressed, with the FMT treatment and oral OC groups showing reductions of approximately 83% and 80%, respectively, relative to the FMT control (*p* < 0.0001).

A similar pattern was observed in female mice (Figure 11D–F). SMYD2 expression was reduced by approximately 68% in the FMT treatment group and 69% in the oral OC group relative to the FMT control, with *p* < 0.0001 for both groups. EZH2 expression was suppressed by approximately 76% and 75% in the FMT treatment and oral OC groups, respectively, compared to the FMT control (*p* < 0.0001).

Notably, the magnitude of SMYD2 and EZH2 suppression was nearly identical for the FMT treatment and oral OC groups across both sexes, indicating that FMT is sufficiently potent to recapitulate the epigenetic suppressive effects of direct oral OC treatments.

## 4. Discussion

CRC remains the second leading cause of cancer-related mortality in the United States, and activating *KRAS* mutations are among the most clinically consequential molecular alterations in this disease, occurring in 40–50% of cases and driving constitutive activation of MAPK/ERK and PI3K/AKT signaling [1,4,5]. Among individual *KRAS* variants, the *G13D* mutation is of particular clinical interest because it can exhibit differential responsiveness to anti-EGFR therapy compared with other *KRAS*-mutant subtypes [7], underscoring the need for improved therapeutic strategies. *KRAS*-mutant tumors are frequently refractory to anti-EGFR-targeted agents and associated with more aggressive disease behavior [5]. The present study aimed to validate OC anti-*KRAS*^G13D^-mutant CRC efficacy in an orthotopic xenografted aggressive *KRAS*^G13D^-mutant HCT-116-Luc model that mimics the clinical tumor microenvironment. This study also intended to determine whether the GM plays a causal role in the anti-CRC activity of OC and assess the direct in vivo efficacy of FMT from highly OC-dosed donors, in comparison to direct oral OC. Two complementary experimental strategies were employed: (i) pharmacological depletion of GM using a non-systemically absorbable broad-spectrum antibiotics cocktail ABC administered alongside oral or intraperitoneal OC to investigate whether GM is required for OC’s full anti-CRC activity, and (ii) FMT using fresh feces from high-dose OC-treated donor mice in recipient nude mice with pre-depleted GM using ABC a week ahead to assess the direct anti-CRC efficacy of OC-modulated GM versus standard oral OC.

A notable strength of the present study is the use of an orthotopic intra-cecally xenografted model, extending our previous findings that OC suppressed *KRAS*^G13D^-mutant CRC progression through the SMYD2–EZH2/c-MET axis in subcutaneously xenografted nude mice [8]. Unlike subcutaneous xenografts, orthotopic xenografting developed CRC within the native colonic microenvironment and remained in direct proximity to GM and their metabolites, making this model particularly reflective of clinical CRC biology [38,39,40,41]. Since OC was previously shown to inhibit the cell-free phosphorylation of c-MET kinase in the Z’-LYTE™ assay, with in vitro and in vivo activation [30], the current study excluded c-MET and focused on assessing the molecular levels of the epigenetic–oncogenic drivers SMYD2 and EZH2. The current orthotopic in vivo model assessment findings not only validate the reproducibility of OC-mediated SMYD2–EZH2 suppression [8] across distinct experimental models for *KRAS*^G13D^-mutant CRC but also show evidence for SMYD2–EZH2 direct downregulation by OC-modulated FMT.

Initial findings of this study revealed markedly greater anti-*KRAS*^G13D^-mutant CRC efficacy with daily oral 10 mg/kg OC rather than with intraperitoneal administration at 10 mg/kg, 3x/week. This observation is particularly noteworthy because intraperitoneal delivery bypasses intestinal–GM interactions and first-pass hepatic metabolism and is expected to provide comparable or even greater efficacy if OC acts primarily through direct effects on tumor cells. Instead, oral OC reduced total tumor weight by 97.6% and 99.0% in male and female mice, respectively, whereas intraperitoneal OC dosing achieved reductions of 64.6% and 69.9% (Table 1). A similar trend was observed for metastatic spread of the *KRAS*^G13D^-mutant CRC HCT-116-Luc, which is a documented model cell line for intestine-to-liver CRC metastasis [41]. Daily oral OC treatments provided near-complete metastasis prevention, unlike ip treatments, which failed to prevent the substantial dissemination of tumor cells. Thus, OC interactions in the intestine via the oral route confer greater anti-CRC efficacy.

If the higher efficacy of oral OC was mostly driven by cross-interaction with GM in the intestine, complete ablation of the GM by the antibiotic cocktail ABC would be expected to reduce oral OC’s efficacy to a level approaching that of the intraperitoneal route. However, the OC + ABC group retained substantial anti-tumor activity that remained far greater than OC ip, and only modestly inferior to oral OC alone (Table 1). A similar pattern was apparent in the metastasis data, in which OC + ABC nearly abolished the tumor metastatic spread to most organs in both sexes. These findings are likely to reflect incomplete GM depletion by the single daily dose of ABC due to the rapid ability of GM to replicate. The single daily ABC dose was intended to minimize animal stress over the experiment course. Double or triple ABC dosing would have ensured complete GM eradication and subsequent accurate assessment of the GM contribution to OC anti-CRC efficacy. However, additional contributions from the direct effects of OC or its final metabolism product tyrosol, residual innate immunity, antibiotic-resistant bacteria, or altered intestinal physiology cannot be excluded. Overall, the partial reduction in efficacy after ABC treatment, together with the robust in vivo effects of FMT, and notably reduced efficacy of OC ip route suggests that the OC-modulated GM or OC final metabolites can potentiate OC-mediated anti-CRC effects.

Longitudinal animal body weight monitoring in the orthotopic intra-cecally xenografted model provided an additional indicator of the overall disease burden and therapeutic response. In the ABC study, the placebo control-treated mice exhibited notable progressive weight loss, consistent with tumor-associated cachexia, whereas oral OC and OC + ABC largely preserved animals’ body weight throughout the study. In contrast, mice treated with ip OC showed a modest decline in weight, consistent with reduced anti-CRC efficacy (Figure 2C and Figure 3C). A similar pattern was also observed in the FMT study, where FMT control mice experienced substantial weight loss, unlike mice treated with OC-dosed donors FMT and oral OC-treated groups, which maintained a stable body weight (Figure 8C and Figure 9C).

While the ABC experiments established partial GM contribution to the anti-CRC activity of oral OC, the FMT study demonstrated significant therapeutic efficacy. These findings indicate that the anti-CRC activity of OC is largely transferable through fresh feces, highlighting the need for future comprehensive studies. Consistent with previous reports showing that FMT suppressed CRC by restoring microbial homeostasis and enhancing anti-tumor immunity [19,22], the findings of this study further suggest that donor GM can be deliberately optimized through dietary interventions. Unlike current microbiota-based approaches that rely on naturally protective bacterial taxa, such as *Roseburia intestinalis* or *Bifidobacterium animalis* [21,27,28], the OC-conditioned fresh feces generate a novel potential approach for CRC control.

SMYD2 is frequently dysregulated in CRC and other gastrointestinal cancers, where it promotes tumor progression and chemoresistance [8,9,10,11,12,13,14]. Likewise, EZH2 is highly expressed in CRC and contributes to its invasion, metastasis, and immune regulation [15,16]. SMYD2 and EZH2 expressions followed the same order as tumor and metastatic burden in both sexes in the ABC study. Oral OC produced the greatest downregulation, OC + ABC afforded intermediate downregulation, and OC ip showed the least expression reduction (Figure 6A,D). The FMT Western blot data reinforced this conclusion.

The consistent therapeutic response observed in both sexes suggests that the anti-CRC effects of oral OC are unlikely to be driven by sex-specific hormonal or metabolic differences. Instead, these findings support a broadly applicable therapeutic benefit across sexes, enhancing the translational relevance of this approach.

The 10 mg/kg oral OC dose that conferred the significant anti-CRC and antimetastatic effects in this study can be translated to a human-equivalent dose. Using the standard 0.081 Km mouse-to-human conversion factor, this works out to approximately 0.81 mg/kg [42], or roughly 56.7 mg of OC per day for an average 70 kg adult. High-phenolic EVOO typically contains approximately 1000 mg OC/L [37]. This corresponds to a daily intake of approximately 56.7 mL of OC-rich EVOO, which is a quantity well within realistic dietary consumption ranges. These calculations place the present findings within a translationally feasible range, supporting oral OC use, whether as a purified nutraceutical or through high-phenolic EVOO, for future anti-CRC clinical trials.

Although the estimated human-equivalent dose provides a preliminary translational perspective, several factors should be considered for clinical dose calculations. The present study evaluated purified OC administered by oral gavage, whereas dietary intake can occur within the complex EVOO matrix, the monounsaturated lipids and accompanying minor phenolics of which may influence OC bioavailability and biological activity. Furthermore, human pharmacokinetics data for OC are currently unavailable, and limited preclinical pharmacokinetics evidence is insufficient to predict human absorption, metabolism, and systemic exposure. Therefore, the translational relevance of this study’s findings should be considered preliminary until validated in future human pharmacokinetic and clinical studies.

The acute and chronic safety profiles of OC further support its translational potential. A single-acute oral dose of OC at 10, 250, and 500 mg/kg produced no mortality in Swiss albino mice, establishing an oral LD_50_ greater than 500 mg/kg and an OECD class 4 toxicity ranking, which is substantially safer than the intraperitoneal route, for which the LD_50_ value ranged from 164 to 524 mg/kg, further highlighting the positive role of GM in OC safety and therapeutic profiles [43]. This favorable oral safety margin reinforces the present study’s central finding that the oral route is not only more efficacious but also better tolerated than parenteral OC administration. Chronic dosing studies in 5xFAD mice indicate that OC is generally non-toxic at 5 and 10 mg/kg but produces hepatotoxicity at 20 mg/kg with prolonged 4-month administration [44].

## 5. Conclusions

Collectively, the study results validate OC as a lead suppressor for *KRAS*^G13D^-mutant CRC progression and metastasis. The study provides a compelling translational and therapeutic rationale to continue development of oral OC and OC-modulated fecal GM as potential interventions for *KRAS*^G13D^-mutant CRC.

## Figures and Tables

**Table 1:** Total primary tumor weights (mg) in male and female nude mice for the ABC study.

Group	Male Tumor Weight, mg	Female Tumor Weight, mg
dpEVOO + ABC, oral	7404.5	6540.1
OC 10 mg/kg, oral	178.4	62.6
OC 10 mg/kg + ABC, oral	212.8	262.6
OC 10 mg/kg, ip, 3×/week	2617.7	1968.0

**Table 2:** FMT study of total primary tumor weights (mg) in male and female mice.

Group	Male Tumor Weight, mg	Female Tumor Weight, mg
FMT Control	9252.2	6594.8
FMT Treatment	48.5	38.4
OC, oral 10 mg/kg	211.2	186.8

## Data Availability

All data used to support the findings of this study have been made available in this publication as figures, tables, or Supplementary Materials.

## Supplementary Materials

The following supporting information can be downloaded at https://www.mdpi.com/article/10.3390/nu18162623/s1. Figure S1: Representative bioluminescence images of metastatic dissemination in major organs for ABC study groups; Figure S2: Raw Western blot images demonstrate the effect of OC and OC-modulated GM on SMYD2, EZH2; Figure S3: Representative bioluminescence images of metastatic dissemination in major organs for FMT study groups; Table S1: Individual primary tumor weights in mg across the treatment groups; Table S2: Individual body weight measurements of mice in grams at different time points across the treatment groups; Table S3: Individual tumor bioluminescence average radiance (photons/s/cm^2^/sr) values for male and female mice across all treatment groups in the ABC study; Table S4: Individual Western blot densitometry values for different treatment groups; Table S5: Individual tumor bioluminescence average radiance (photons/s/cm^2^/sr) values for male and female mice across all treatment groups in the FMT study.
