# Supplementary material for: Lead Validation of the Olive Phenolic S-(−)-Oleocanthal for Effective Control of KRASG13D-Mutant Colorectal Cancer Progression and Metastasis
Source: Nutrients. 2026 Aug 11;18(16):2623. doi: 10.3390/nu18162623 (PMC13516816; doi:10.3390/nu18162623)
Supplement: Supplementary file 1 [file nutrients-18-02623-s001.zip › nutrients-4445593-supplementary.pdf]

nutrients

MDPI

Supplementary Materials

Lead Validation of the Olive Phenolic *S*-(-)-Oleocanthal for Effective Control of *KRAS*^{G13D}-Mutant Colorectal Cancer Progression and Metastasis

Md Towhidul Islam Tarun [1], Hassan Y. Ebrahim [2], Khalid A. El Sayed [1,*]

[1] School of Basic Pharmaceutical and Toxicological Sciences, College of Pharmacy, University of Louisiana at Monroe, 1800 Bienville Drive, Monroe, LA 71201, USA; tarunmt@warhawks.ulm.edu (M.T.T.).
[2] Department of Biomedical Sciences, Discipline of Pharmacology, Edward Via College of Osteopathic Medicine, Monroe, LA 71203, USA; hebrahim@ulm.vcom.edu (H.Y.E.).

* Correspondence: elsayed@ulm.edu (K.A.E.); Tel.: +1-318-342-1725

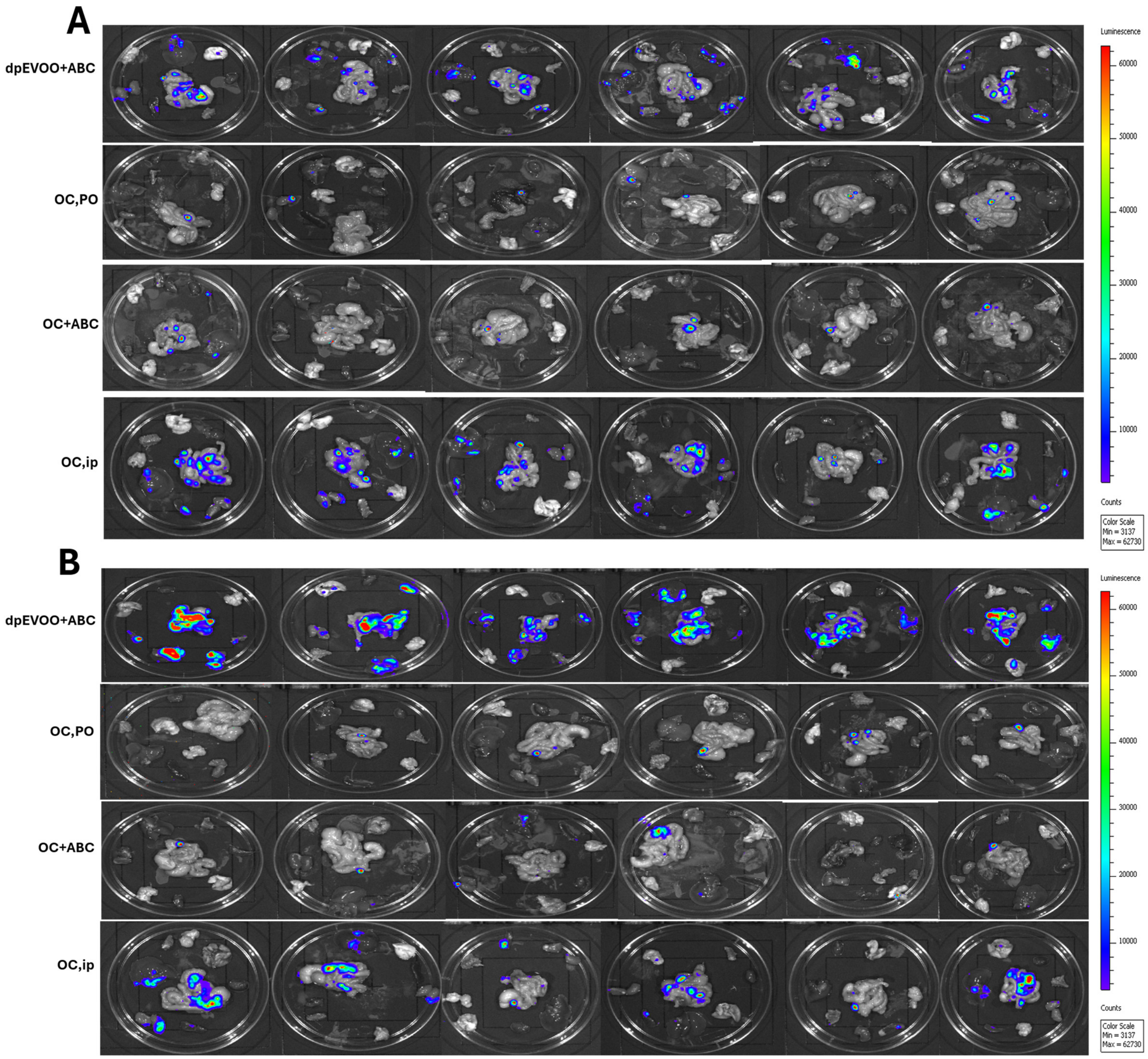

Figure S1. Representative bioluminescence images of metastatic dissemination in major organs from (A) Male and (B) Female orthotopic HCT-116-Luc CRC-bearing nude mice following ABC study groups.

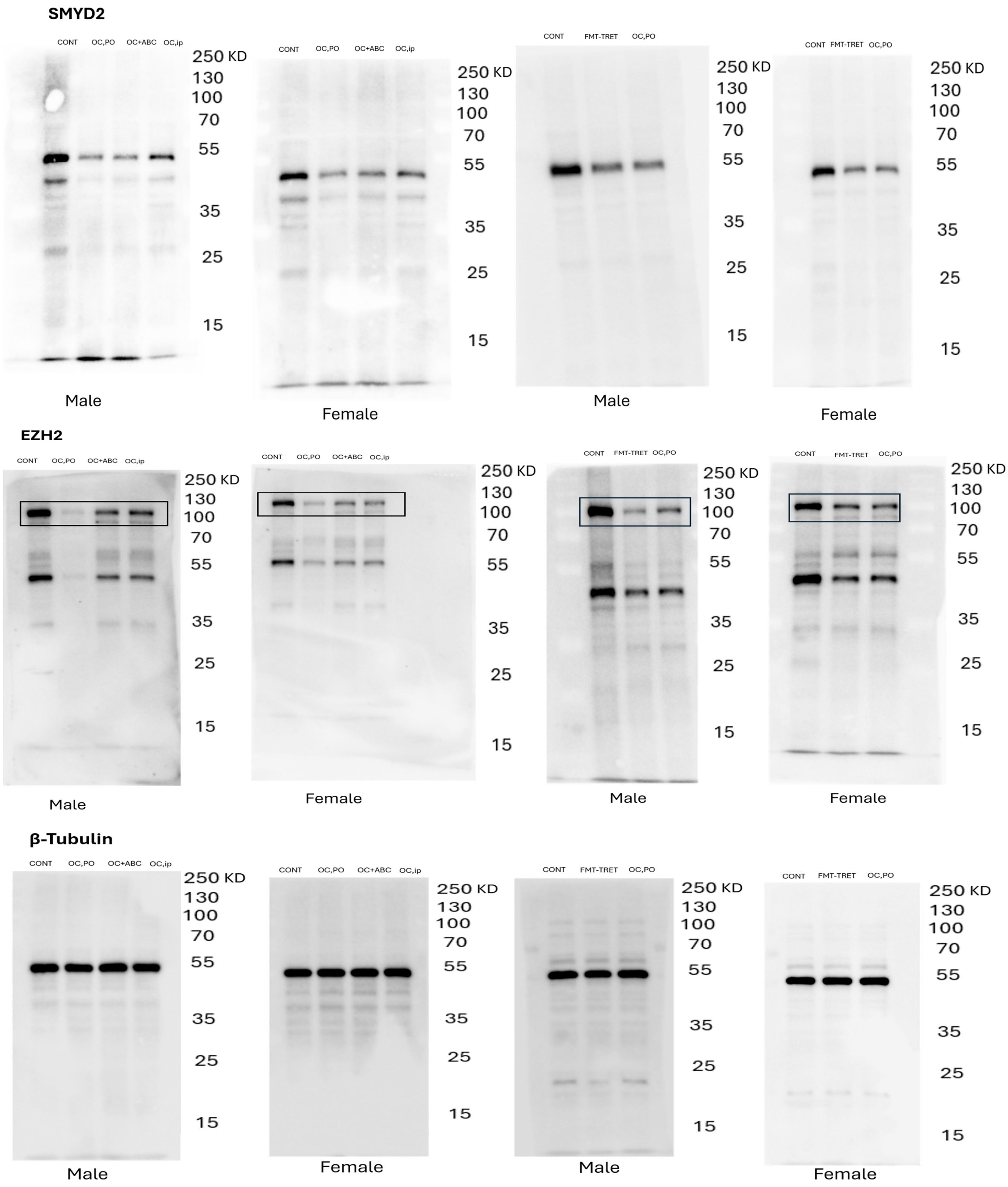

Figure S2. Raw Western blot images demonstrate the effect of OC and OC modulated GM on SMYD2, EZH2. β-Tubulin used as loading control.

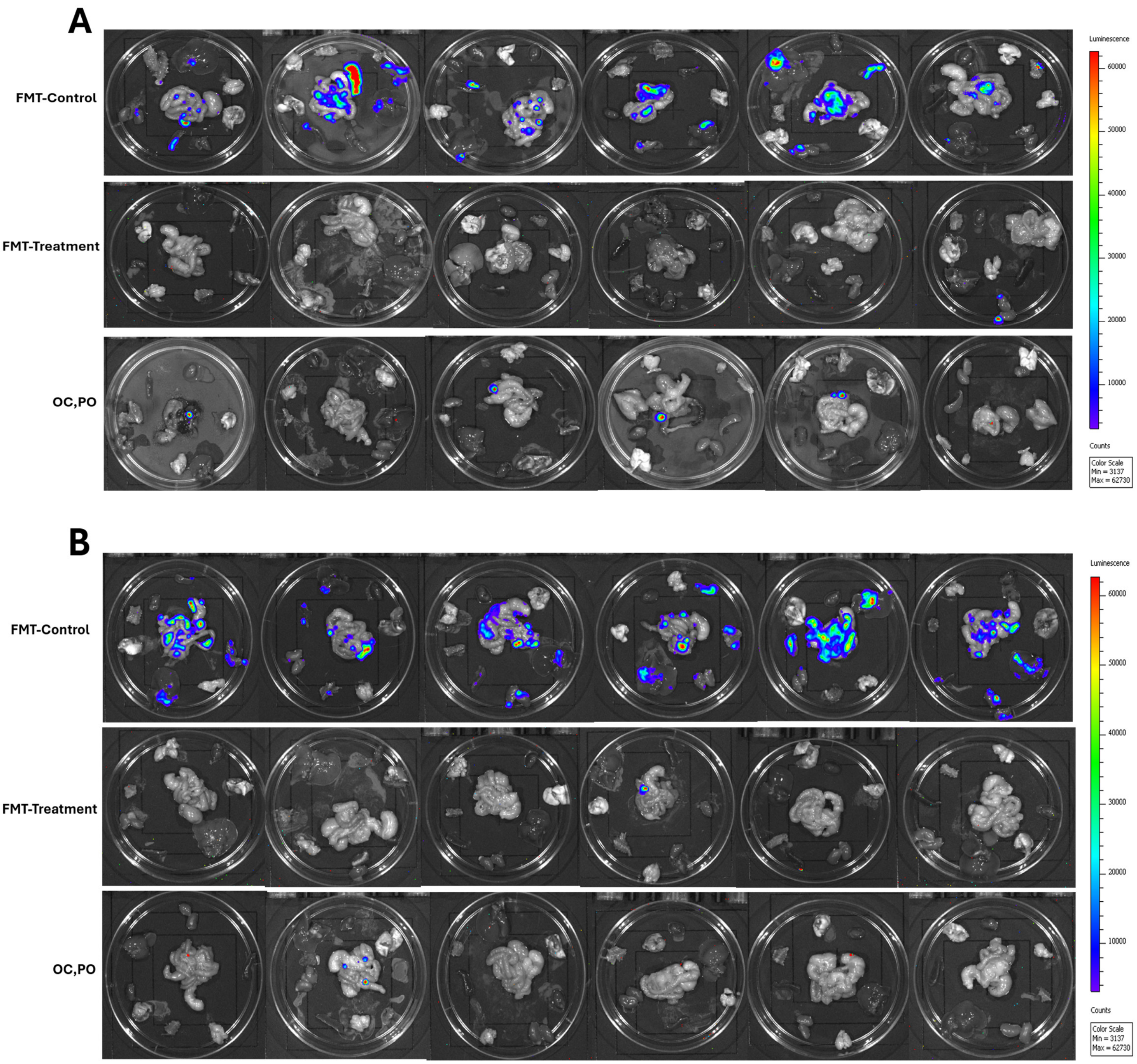

Figure S3. Representative bioluminescence images of metastatic dissemination in major organs from (A) Male and (B) Female orthotopic HCT-116-Luc CRC-bearing nude mice following FMT study groups.

Table S1. Individual primary tumor weights in mg across the treatment groups.

A. Male mice (ABC study)

Mouse	dpEVOO+ABC (Control)	OC, PO	OC+ABC	OC, ip
**Mouse 1**	1183.2	24.8	97.2	429
**Mouse 2**	1878.7	10.4	9.7	428.5
**Mouse 3**	955	69.9	30.5	358.7
**Mouse 4**	1083.3	43	17.5	521.6
**Mouse 5**	1205.3	9	47.5	437.7
**Mouse 6**	1099	3.1	10.4	442.2
**Mouse 7**	—	5.6	—	—
**Mouse 8**	—	12.6	—	—

B. Female mice (ABC study)

Mouse	dpEVOO+ABC (Control)	OC, PO	OC+ABC	OC, ip
**Mouse 1**	717.4	29.1	31.1	223.1
**Mouse 2**	1380.5	17.5	30	428
**Mouse 3**	850	3	111.9	399.4
**Mouse 4**	1547.9	5	58.9	256.3
**Mouse 5**	608.3	3	20.4	309
**Mouse 6**	1436	5	10.3	352.2

C. Male mice (FMT study)

Mouse	FMT Control (n=6)	FMT Treatment (n=6)	OC, PO (n=6)
**Mouse 1**	1745.3	20.1	22.2
**Mouse 2**	1801.5	11	55.2
**Mouse 3**	1445.2	4.2	50.4
**Mouse 4**	1523.1	5	48.3
**Mouse 5**	1498.4	5	20.1
**Mouse 6**	1238.7	3.2	15

D. Female mice (FMT study)

Mouse	FMT Control (n=6)	FMT Treatment (n=6)	OC, PO (n=6)
**Mouse 1**	1534.2	10	45.3
**Mouse 2**	1267	12.2	40.2
**Mouse 3**	1133.3	5.2	38.4
**Mouse 4**	988.8	3	37.3
**Mouse 5**	786.3	5	20.1
**Mouse 6**	885.2	3	5.5

Table S2. Individual body weight measurements of mice in gram at different time points across the treatment groups.

A. Male mice (ABC study)

Day	dpEVOO+ABC (Control)	OC, PO	OC+ABC	OC, ip
**3**	24.5, 25.6, 23.4, 23.8, 26.6, 27.7	23.6, 24.6, 26.4, 22.8, 22.9, 23.9	23.3, 23.5, 23.7, 23.2, 25.8, 26.5	26.7, 22.8, 23.8, 25.8, 22.5
**7**	24.0, 25.0, 23.1, 23.5, 26.5, 27.5	23.7, 24.6, 26.4, 22.8, 23.0, 23.9	23.4, 23.5, 23.7, 23.2, 26.0, 26.5	26.8, 22.8, 23.8, 25.8, 22.5
**10**	23.5, 24.4, 22.5, 23.0, 25.2, 26.6	23.7, 24.6, 26.5, 22.9, 23.0, 24.0	23.5, 23.5, 23.7, 23.2, 26.1, 26.5	26.0, 22.4, 23.8, 25.8, 22.0
**14**	23.0, 24.2, 22.0, 21.0, 24.8, 25.0	23.8, 24.7, 26.5, 23.0, 23.0, 25.2	23.9, 23.7, 24.1, 23.2, 26.2, 26.6	25.5, 22.4, 23.7, 25.8, 22.0
**17**	22.0, 22.0, 21.2, 20.2, 23.0, 22.8	23.7, 24.7, 26.8, 23.0, 23.2, 25.2	23.8, 23.8, 24.2, 23.2, 26.2, 26.7	24.0, 20.3, 23.7, 25.9, 22.0
**21**	20.0, 20.2, 19.0, 18.9, 22.6, 22.2	23.8, 24.7, 26.8, 23.2, 23.2, 25.2	23.9, 23.8, 24.5, 23.2, 26.2, 26.7	24.0, 20.3, 23.6, 25.8, 22.2

B. Female mice (Antibiotic study)

Day	dpEVOO+ABC (Control)	OC, PO	OC+ABC	OC, ip
**3**	23.4, 23.6, 22.5, 26.6, 27.7, 25.5	23.5, 23.6, 26.5, 24.7, 22.8, 25.9	23.3, 23.4, 23.2, 23.2, 25.6, 27.0	26.6, 22.7, 23.5, 25.6, 22.5, 23.9
**7**	23.0, 23.2, 22.4, 26.0, 26.5, 25.2	23.5, 23.6, 26.5, 24.8, 22.9, 25.9	23.4, 23.5, 23.7, 23.2, 26.0, 26.9	26.8, 22.8, 23.8, 25.7, 22.5, 23.8

Day	dpEVOO+ABC (Control)	OC, PO	OC+ABC	OC, ip
**10**	23.2, 22.5, 21.2, 25.0, 26.0, 25.2	23.5, 23.8, 26.8, 24.8, 22.9, 26.0	23.5, 23.5, 23.7, 23.0, 26.1, 27.1	26.0, 22.5, 23.6, 24.4, 22.0, 23.9
**14**	22.5, 22.1, 20.4, 24.2, 25.9, 24.1	23.4, 23.8, 26.8, 24.8, 22.8, 26.2	23.9, 23.7, 24.1, 23.0, 26.2, 27.0	25.5, 21.0, 22.7, 23.0, 22.0, 22.7
**17**	22.0, 19.5, 19.7, 23.5, 24.3, 23.8	23.6, 23.9, 26.9, 25.0, 22.9, 26.2	23.4, 23.7, 24.1, 23.5, 26.0, 26.8	25.0, 21.0, 22.7, 23.0, 21.9, 22.5
**21**	20.0, 19.0, 17.8, 22.0, 24.0, 22.2	23.6, 24.0, 27.2, 25.2, 22.9, 26.3	23.5, 23.7, 24.2, 23.5, 26.1, 27.0	25.0, 21.4, 22.5, 23.2, 21.8, 22.5

C. Male mice (FMT study)

Day	FMT Control (*n*=6)	FMT Treatment (*n*=6)	OC, PO (*n*=6)
**3**	23.0, 25.5, 25.7, 24.8, 22.5, 23.5	23.4, 23.2, 23.8, 25.0, 26.6, 24.4	24.7, 23.7, 24.3, 23.5, 23.8, 23.1
**7**	23.0, 24.8, 25.5, 23.6, 21.0, 22.0	23.4, 23.2, 23.8, 25.0, 26.6, 24.4	24.7, 23.7, 24.3, 23.5, 23.8, 23.1
**10**	21.0, 22.5, 24.7, 21.5, 20.0, 21.0	23.3, 23.3, 23.8, 25.1, 26.7, 24.4	24.9, 23.7, 24.3, 23.9, 23.7, 23.1
**14**	19.6, 22.0, 24.0, 21.1, 19.0, 20.0	23.6, 23.4, 23.8, 25.3, 26.9, 24.5	25.2, 23.7, 24.5, 23.9, 23.9, 23.1
**17**	18.0, 21.0, 23.8, 21.0, 18.0, 19.0	24.0, 23.8, 23.9, 25.6, 27.0, 24.6	25.3, 23.8, 24.5, 24.2, 24.4, 23.0
**21**	17.0, 20.0, 22.0, 19.5, 17.0, 18.0	24.1, 24.0, 24.3, 25.6, 27.0, 24.6	25.3, 24.0, 24.5, 24.4, 24.3, 23.5

D. Female mice (FMT study)

Day	FMT Control (*n*=6)	FMT Treatment (*n*=6)	OC, PO (*n*=6)
**3**	27.5, 22.5, 23.8, 23.7, 20.8, 26.8	24.3, 22.9, 22.8, 27.5, 25.2, 23.0	23.5, 23.1, 22.7, 27.5, 26.5, 24.2
**7**	27.0, 22.1, 23.8, 23.5, 20.1, 26.5	24.3, 23.0, 22.8, 27.5, 25.3, 23.0	23.5, 23.1, 22.7, 27.5, 26.5, 24.2
**10**	26.1, 21.7, 23.0, 23.1, 19.5, 26.1	24.4, 23.0, 22.8, 27.7, 25.5, 23.0	23.5, 23.5, 23.0, 27.7, 26.9, 24.2
**14**	25.5, 21.0, 21.0, 21.0, 18.2, 25.0	24.3, 23.0, 22.9, 27.9, 25.5, 23.5	23.6, 23.5, 23.0, 27.7, 26.9, 24.5
**17**	24.7, 19.0, 19.0, 20.5, 17.0, 24.0	24.4, 23.1, 22.9, 28.2, 25.5, 23.5	23.6, 23.4, 23.0, 27.8, 27.1, 24.5
**21**	24.2, 17.5, 18.7, 19.2, 17.0, 23.2	24.4, 23.2, 22.9, 28.2, 25.5, 23.8	23.6, 23.5, 23.2, 27.8, 27.1, 24.5

Table S3. Individual tumor bioluminescence average radiance (photons/s/cm²/sr) values for male and female mice across all treatment groups in ABC study.

Sex	dpEVOO+ABC	OC, PO	OC+ABC	OC, ip
Male	7.62×10^8	7.83×10^7	1.19×10^8	2.43×10^8
	4.73×10^8	5.24×10^7	2.65×10^7	9.84×10^7
	2.16×10^9	5.70×10^7	2.19×10^7	5.12×10^8
	9.93×10^8	1.04×10^7	2.67×10^7	7.83×10^7
	1.17×10^9	1.92×10^6	1.55×10^8	3.38×10^8
	1.16×10^9	1.04×10^7	7.40×10^7	2.21×10^8
Female	3.12×10^9	9.13×10^6	5.70×10^7	1.19×10^8
	2.86×10^9	1.29×10^7	2.23×10^7	2.43×10^8
	1.34×10^9	5.14×10^7	3.27×10^8	2.34×10^8
	1.12×10^9	9.16×10^7	1.29×10^7	4.85×10^8
	1.20×10^9	5.63×10^6	2.34×10^7	6.84×10^8
	1.00×10^9	1.32×10^7	1.27×10^7	7.62×10^8

Table S4. Individual Western Blot densitometry values for different treatment groups.

A. Male mice (ABC study)

Group	SMYD2-1	SMYD2-2	SMYD2-3	EZH2-1	EZH2-2	EZH2-3
**dpEVOO+ABC**	1.10	0.97	0.90	1.10	0.97	0.90
**OC, PO**	0.32	0.45	0.29	0.06	0.04	0.23
**OC+ABC**	0.36	0.48	0.32	0.29	0.32	0.32
**OC, ip**	0.54	0.52	0.31	0.42	0.26	0.57

B. Female mice (ABC study)

Group	SMYD2-1	SMYD2-2	SMYD2-3	EZH2-1	EZH2-2	EZH2-3
**dpEVOO+ABC**	1.00	1.10	1.09	1.00	1.10	1.09
**OC, PO**	0.27	0.30	0.43	0.13	0.19	0.15
**OC+ABC**	0.38	0.41	0.54	0.28	0.26	0.14

Group	SMYD2-1	SMYD2-2	SMYD2-3	EZH2-1	EZH2-2	EZH2-3
**OC, ip**	0.42	0.50	0.63	0.25	0.22	0.51

C. Male mice (FMT study)

Group	SMYD2-1	SMYD2-2	SMYD2-3	EZH2-1	EZH2-2	EZH2-3
**Control**	1.00	0.92	1.12	1.00	0.92	1.12
**FMT Treatment**	0.35	0.30	0.35	0.37	0.18	0.22
**OC, PO**	0.32	0.29	0.36	0.34	0.23	0.22

D. Female mice (FMT study)

Group	SMYD2-1	SMYD2-2	SMYD2-3	EZH2-1	EZH2-2	EZH2-3
**Control**	1.00	1.09	0.90	1.00	1.09	0.90
**FMT Treatment**	0.43	0.38	0.39	0.14	0.14	0.22
**OC, PO**	0.37	0.41	0.35	0.21	0.16	0.22

Table S5. Individual tumor bioluminescence average radiance (photons/s/cm²/sr) values for male and female mice across all treatment groups in FMT study.

Sex	FMT Cont	FMT Treatment	OC, PO
Male	2.18×10^9	8.90×10^6	1.18×10^8
	1.95×10^9	1.26×10^7	1.42×10^8
	1.67×10^9	1.74×10^7	1.67×10^8
	8.81×10^8	2.08×10^7	1.93×10^8
	2.19×10^9	2.53×10^7	2.21×10^8
	1.98×10^9	3.02×10^7	2.48×10^8
Female	7.94×10^8	7.80×10^6	1.09×10^8
	2.71×10^9	1.11×10^7	1.31×10^8
	2.48×10^9	1.59×10^7	1.56×10^8
	2.22×10^9	1.98×10^7	1.84×10^8
	2.06×10^9	2.37×10^7	2.12×10^8
	1.87×10^9	2.28×10^8	2.39×10^8
